# Chromosome Diversity and Evolution in Helicoide a (Gastropoda: Stylommatophora): A Synthesis from Original and Literature Data

**DOI:** 10.3390/ani11092551

**Published:** 2021-08-30

**Authors:** Agnese Petraccioli, Paolo Crovato, Fabio Maria Guarino, Marcello Mezzasalma, Gaetano Odierna, Orfeo Picariello, Nicola Maio

**Affiliations:** 1 Department of Biology, University of Naples Federico II, I-80126 Naples, Italy; petra.ag@gmail.com (A.P.); fabio.guarino@unina.it (F.M.G.); orfeo.picariello@unina.it (O.P.); nicomaio@unina.it (N.M.); 2 Società Italiana di Malacologia, Via Mezzocannone, 8-80134 Naples, Italy; paolo.crovato@fastwebnet.it; 3 CIBIO-InBIO, Centro de Investigação em Biodiversidade e Recursos Genéticos, InBIO, Universidade do Porto, Rua Padre Armando Quintas 7, 4485-661 Vairaõ, Portugal

**Keywords:** 16S rRNA gene, evolution, FISH, karyotype, molecular phylogeny, mollusca

## Abstract

**Simple Summary:**

The superfamily Helicoidea is a large and diverse group of Eupulmonata. The superfamily has been the subject of several molecular and phylogenetic studies which greatly improved our knowledge on the evolutionary relationships and historical biogeography of many families. In contrast, the available karyological information on Helicoidea still results in an obscure general picture, lacking a homogeneous methodological approach and a consistent taxonomic record. Nevertheless, the available karyological information highlights the occurrence of a significant chromosomal diversity in the superfamily in terms of chromosome number (varying from 2*n* = 40 to 2*n* = 62), chromosome morphology and the distribution of different karyological features among different taxonomic groups. Here we performed a molecular and a comparative cytogenetic analysis on of 15 Helicoidea species of three different families. Furthermore, to provide an updated assessment of the chromosomal diversity of the superfamily we reviewed all the available chromosome data. Finally, superimposing all the chromosome data gathered from different sources on the available phylogenetic relationships of the studied taxa, we discuss the overall observed chromosome diversity in Helicoidea and advance a hypothesis on its chromosomal evolution.

**Abstract:**

We performed a molecular and a comparative cytogenetic analysis on different Helicoidea species and a review of all the available chromosome data on the superfamily to provide an updated assessment of its karyological diversity. Standard karyotyping, banding techniques, and Fluorescence in situ hybridization of Nucleolus Organizer Region loci (NOR-FISH) were performed on fifteen species of three families: two Geomitridae, four Hygromiidae and nine Helicidae. The karyotypes of the studied species varied from 2*n* = 44 to 2*n* = 60, highlighting a high karyological diversity. NORs were on a single chromosome pair in *Cernuella virgata* and on multiple pairs in four Helicidae, representing ancestral and derived conditions, respectively. Heterochromatic C-bands were found on pericentromeric regions of few chromosomes, being Q- and 4′,6-diamidino-2-phenylindole (DAPI) negative. NOR-associated heterochromatin was C-banding and chromomycin A_3_ (CMA_3_) positive. Considering the available karyological evidence on Helicoidea and superimposing the chromosome data gathered from different sources on available phylogenetic inferences, we describe a karyotype of 2*n* = 60 with all biarmed elements as the ancestral state in the superfamily. From this condition, an accumulation of chromosome translocations led to karyotypes with a lower chromosome number (2*n* = 50–44). This process occurred independently in different lineages, while an augment of the chromosome number was detectable in Polygyridae. Chromosome inversions were also relevant chromosome rearrangements in Helicoidea, leading to the formation of telocentric elements in karyotypes with a relatively low chromosome count.

## 1. Introduction

The land snails of the superfamily Helicoidea include about 5600 species, constituting a large and diverse group of the about 25,000 Eupulmonata so far described [1,2]. The superfamily has an almost worldwide distribution, being absent only in sub-Saharan continental Africa, southern South America, some Pacific islands, and New Zealand [3,4]. The complex classification and taxonomy of the Helicoidea have been revised several times [5,6,7,8,9], and the species of the superfamily are currently distributed in 16 families, 37 subfamilies, and 359 genera [1].

Helicoidea, due to their peculiar natural history and historical biogeography, are interesting models for studies on evolutionary dynamics, and recent molecular works have started to provide more accurate representations of their evolutionary relationships (e.g., [10,11,12,13]). This is particularly true for several families and subfamilies, whose phylogenetic relationships have been described in several focused works (e.g., [10,13,14]), and more in general for the Helicoidea of the Western Palearctic, whose classification and phylogeny have been recently revised [9]. In particular, Razkin et al. [9] proposed an updated classification and phylogenetic relationships of the western Palearctic Helicoidea, confirming the taxonomic validity of many morphologically defined families and re-defining the systematic boundaries of many different groups respecting the monophyly of families, subfamilies, and tribes [9]. In addition, the origin of the whole superfamily Helicoidea was estimated in the early Cretaceous period, while its families were estimated to be from Late-Cretaceous to Paleogene period [9].

In contrast to a progressively clearer phylogenetic scenario emerging from recent molecular studies, the available karyological information on the superfamily is scattered among older and more recent papers, lacking a homogeneous methodological approach and a consistent taxonomic record, and thus resulting in an obscure general picture. Nevertheless, the available karyological information highlights the occurrence of a significant chromosomal diversity in the superfamily in terms of chromosome number (varying from 2*n* = 40 to 2*n* = 62), chromosome morphology, and the distribution of different karyological features among different taxonomic groups (see e.g., [15,16]).

Historically, two different reviews have summarized chromosome information on mollusks in general [17] and gastropods [16], helping to elucidate their overall karyological diversity. However, in Patterson [17] there was some missing information concerning previously described karyotypes of camenids and polygirids (see [18,19]), while in Thiriot-Quiévreux [16], which included published karyological information from Patterson [17] in 2002, missing data involve several different evolutionary lineages (see [20,21,22,23,24,25]). The whole picture on the available chromosome diversity of the Helicoidea thus appears still incomplete and future research as well as evolutionary inferences on the overall karyological diversity of the subfamily would benefit from a new, updated assessment of the available data. Indeed, karyological data, especially when linked to molecular inferences, can be valuable tools to individuate plesio- and apomorphic states, identify and characterize different evolutionary lineages, and to assess taxonomic uncertainties (see e.g., [26,27,28]). The usefulness of cytogenetic studies in evolutionary and taxonomic inferences has been recently shown in different mollusk taxa, highlighting the main evolutionary events of their karyotype diversification (e.g., [29,30,31]), but they usually involved low level taxonomic groups or just a handful of related species.

In this study, we performed a molecular and a comparative karyological analysis with standard, Quinacrine (Q-) staining, DAPI- and CMA_3_ banding, sequential C banding + fluorochromes, and NOR-FISH on land snail species belonging to three different Helicoidea families (Hygromiidae, Geomitridae, and Helicidae). To provide a molecular taxonomic attribution of the studied specimens, we also performed a molecular analysis using a segment of the mitochondrial 16S rRNA, which has been largely used in previous molecular studies on Helicoidea [9,13,14,32,33,34]. Furthermore, to provide an updated assessment of the chromosomal diversity of the superfamily, we reviewed all the available literature from 1946 to 2021 using an updated taxonomy and nomenclature following World Register of Marine Species (WoRMS) [1] and Mollusca Base [2]. Finally, superimposing all the chromosome data gathered from different sources on the available phylogenetic relationships of the studied taxa, we discuss the overall observed chromosome diversity in the superfamily and in different taxonomic groups and advance a hypothesis on their chromosomal evolution.

## 2. Materials and Methods

### 2.1. Sampling

We analyzed a total of 29 specimens of 15 Helicoidea species, including two Geomitridae, nine Helicidae, and four Hygromiidae. Samples were first morphologically determined using conchological and anatomical characters following different sources [35,36,37,38,39,40,41], and subsequently analyzed by molecular methods as described below. A complete list of the studied samples, sampling localities, and their relative taxonomic attribution after morphological and molecular analyses is reported in Table 1.

For comparative purposes, and to provide an updated evaluation of all the available chromosomal data on Helicoidea, we reviewed all the previously published karyotypes of the superfamily using an updated nomenclature following WoRMS [1] and Mollusca Base [2]. A complete list of all the considered karyotypes, taxonomic attribution, and relative references, including a total of 244 chromosome data for 205 species, 97 genera and 8 families, is provided in Table S1.

### 2.2. Molecular Analysis

DNA was extracted from foot tissue samples following Sokolov [42]. For molecular analysis, we choose the mitochondrial 16S rRNA as the selected genetic marker considering its wide use in previous molecular studies on Helicoidea (e.g., [9,13,14,32,33,34]) and its adequate taxon sampling available on GenBank. A mitochondrial segment of 16S rRNA of about 600 bp was amplified using the primer pair 16Sa (CGCCTGTTTATCAAAAACAT) and 16Sb (CCGGTCTGAAACTCAGATCAGT) [43]. PCR parameters: initial denaturation at 94 °C for 5 min, 36 cycles at 94 °C for 30 s, 50 °C and 72 °C for 45 s followed by a final step at 72 °C for 7 min. Amplicons were sequenced on an automated sequencer ABI 377 (Applied Biosystems, Foster City, CA, USA) using BigDye Terminator 3.1 (ABI). Chromatograms were checked and edited using Chromas Lite 2.3.1 (Technelysium Pty Ltd, Brisbane, Australia) and BioEdit 7.2.6.1 [44]. All the newly determined sequences were deposited in GenBank (accession numbers: MZ504244-MZ504269).

### 2.3. Cytogenetic Analysis

Specimens were injected with colchicine (1 mg/mL; 0.1 mL/10 g body weight) and after three hours were killed by immersion in water. Cell suspensions were obtained from gonads as described in [30]. In brief, the gonads were incubated for 30 min in hypotonic solution (KCl 0.075 M and sodium citrate 0.5% 1:1) and fixed for 15 min in methanol-acetic acid, 3:1. Cells were dissociated manually on a steel sieve and 25 μL of chromosome suspension was sprinkled on the slides. Chromosomes were obtained with the air-drying method [45], stained with traditional 5% Giemsa solution at pH 7 and different other staining and banding techniques: Quinacrine (Q) banding according to Schmid [46], CMA_3_/Methyl green (CMA_3_/MG) according to Sahar and Latt [47], C-banding according to Sumner [48] but performing the denaturation step with Ba(OH)2 for two min at room temperature, and sequential C-banding + Fluorochromes (CMA_3_ + DAPI) [49]. NOR-FISH was performed according to [50], using as a probe the 18S rRNA of the Antarctic scallop *Adamussium colbecki* (Smith, 1902). Ten plates per studied sample were used for karyotype reconstruction and the calculation of relative length (RL) and centromeric index (CI) (Table S2). Chromosome were classified in m = metacentric, sm = submetacentric, st = subtelocentric, and t = telocentric [51].

## 3. Results

### 3.1. Molecular Analysis and Taxonomic Attribution

Successful PCR amplifications were obtained for all the examined specimens except for *Eobania vermiculata* and *Campylaea planospira*, as their DNA were highly degraded. After searches in Basic Local Alignment Search Tool (BLAST) [52], the newly determined 16S sequences showed an identity versus deposited GenBank sequences ranging from 85.9% to 100%, allowing us to provide the final molecular taxonomic attributions reported in Table 1.

### 3.2. Karyotype Description

#### 3.2.1. Family Higromiidae

**Figure 1:**
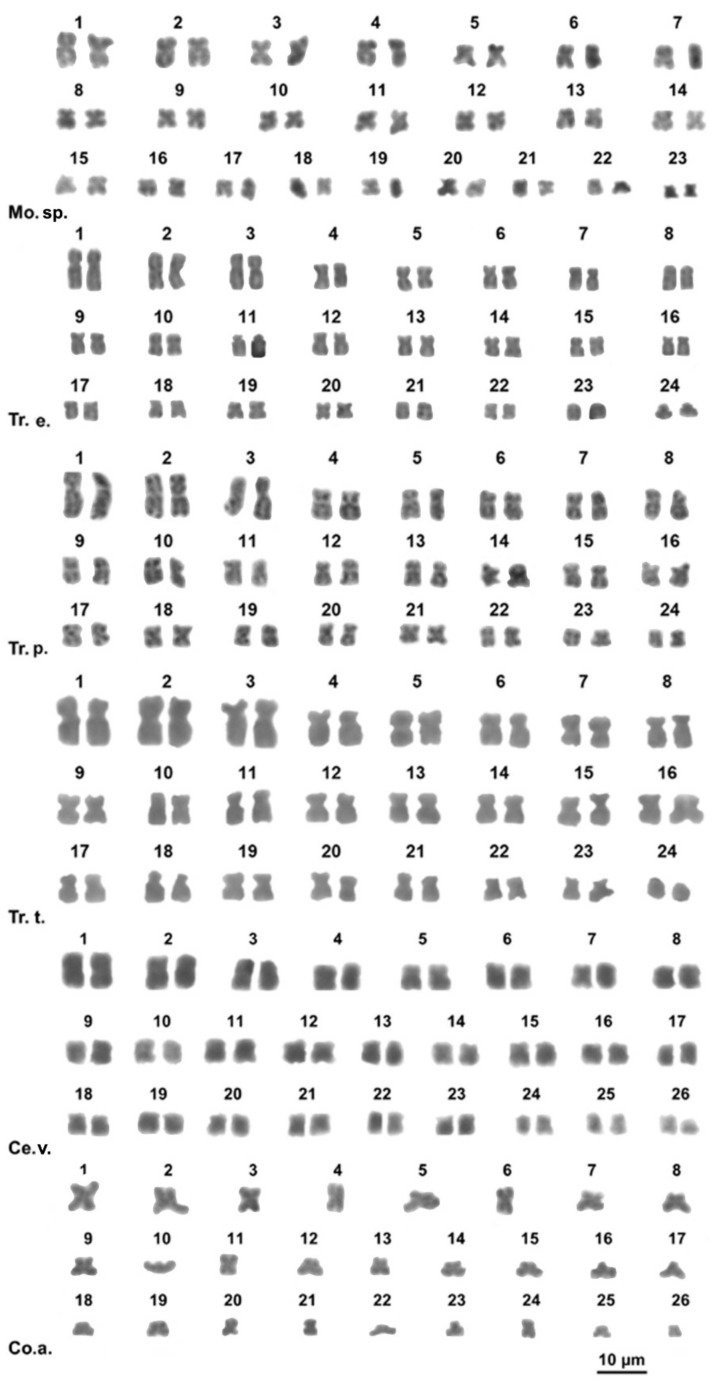
Giemsa stained karyotypes of *Monacha* sp. (Mo.sp.), *Trochoidea elegans* (Tr.e.), *T. pyramidata* (Tr.p.), *T. trochoides* (Tr.t.), *Cernuella virgata* (Ce.v), *Cochlicella acuta* (Co.a.).

The studied *Monacha* sp. specimen had a karyotype of 2*n* = 46 chromosomes gradually decreasing in length. All pairs are metacentric, excluding pairs 2 and 22 that are submetacentric (Figure 1; Table S2).

The three studied *Trochoidea* species (*T. elegans*, *T. pyramidata*, and *T. trochoides*) all showed a karyotype of 2*n* = 48, with mostly biarmed chromosomes and the first three pairs distinctively larger than the other pairs. Nevertheless, the three *Trochoidea* species studied showed a distinct chromosome morphology. In *T. elegans*, the pairs 1, 8–9, 12, 16–17, 21 are submetacentric, the pairs 11 and 23 are telocentric while all the other pairs are metacentric (Figure 1; Table S2). In *Trochoidea pyramidata* the pairs 1, 5 and 11 are submetacentric while all the other pairs are metacentric (Figure 1; Table S2). In *T. trochoides*, the pairs 1, 3, 6, 8, 11 are submetacentric, the pair 24 is telocentric and the remaining pairs are metacentric (Figure 1; Table S2).

#### 3.2.2. Family Geomitridae

Metaphase plates were obtained from specimens of *Cernuella virgata*, while only haploid plates were obtained from *Cochlicella acuta*. *Cernuella virgata* has a karyotype of 2*n* = 52 with all metacentric chromosome pairs, except for the pair 13 and 15 which are submetacentric (Figure 1, Table S2). *Cochlicella acuta* showed a karyotype of *n* = 26 elements; the chromosomes 7–8, 12, 16, 17 and 23 are submetacentric, chromosome 22 is subtelocentric, chromosome 10 is telocentric while all the remaining elements are metacentric (Figure 1, Table S2).

#### 3.2.3. Family Helicidae

**Figure 2:**
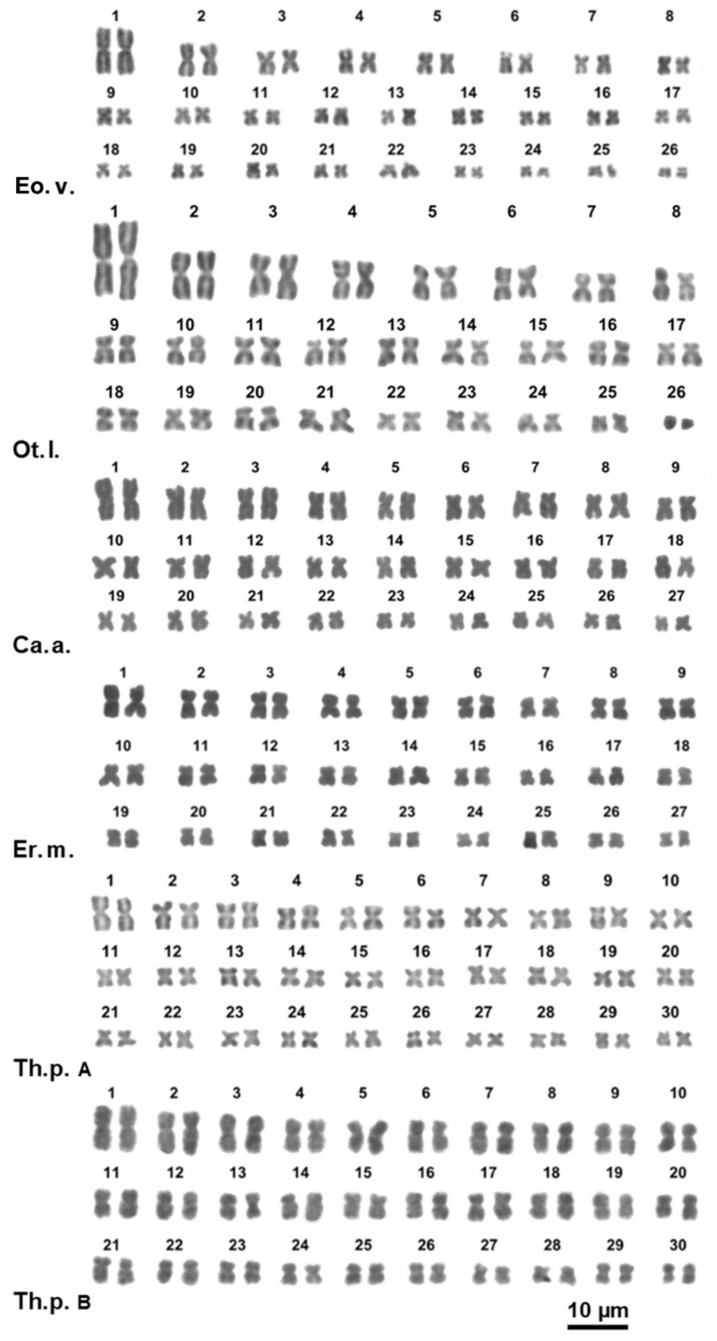
Giemsa-stained karyotypes of *Eobania vermiculata* (Eo.v.), *Otala lactea* (Ot.l.), *Cantareus apertus* (Ca.a.), *Erctella mazzullii* (Er.m.), *Theba pisana* from Fusaro lake (Th.p. A), *Theba pisana* from Messina (Th.p. B).

**Figure 3:**
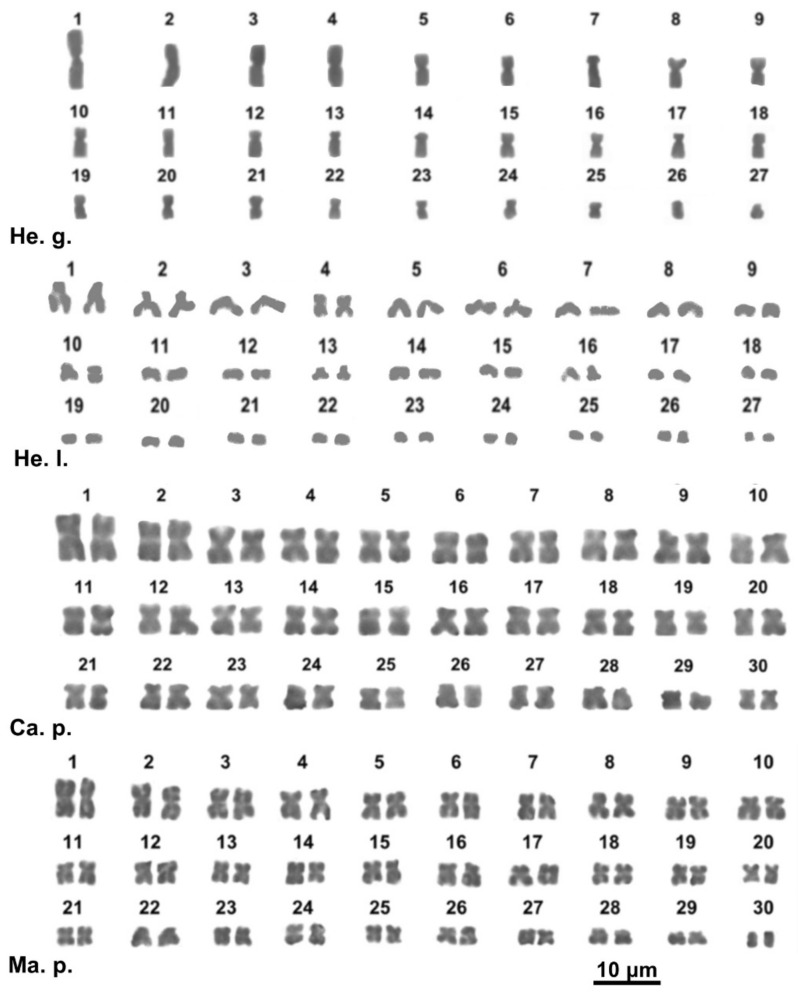
Giemsa-stained karyotypes of *Helix gussoneana* (He. g.), *H. lucorum*. (He. l.), *Campylaea planospira* (Ca. p.), *Marmorana platychela* (Ma. p.).

The eight studied species of Helicidae showed karyotypes from 2*n* = 52 to 2*n* = 60 chromosomes. Variability in the chromosome number was observed both among and within the studied subfamilies and tribes (Table 1; Figure 2 and Figure 3).

*Cornu apertus* and *Erctella mazzullii* showed a karyotype of 2*n* = 54 with mostly metacentric chromosomes, excluding pairs 16 and 20 of *E. mazzullii* and pairs 5, 9, 18, 23 and 25 of *C. apertus* that are submetacentric (Figure 2, Table S2). The elements of the first pair were about 50% larger than those of pairs 2 and the remaining pairs gradually decreased in length (Figure 2; Table S2). The other two examined Otalini species, *Eobania vermiculata* and *Otala lactea*, have a karyotype of 2*n* = 52 chromosomes. In *E. vermiculata* the pairs 12 and 22 are submetacentric while all the remaining pairs are metacentric (Figure 2; Table S2). In *O. lactea* the pairs 8, 9, 12, 17 and 20 are submetacentic, the last pair is telocentric, and all the remaining pairs are metacentric (Figure 2, Table S2). Both species have the chromosomes of the first pair being about 1.8 times larger than the second one, while the remaining pairs gradually decrease in length (Figure 2, Table S2).

The *Theba pisana* specimens from Messina and Fusaro lake showed a karyotype of 2*n* = 60 with all metacentric chromosomes, gradually decreasing in length (Figure 2, Table S2).

The studied specimen of *Helix gussoneana* showed a karyotype of 2*n* = 54 chromosomes, of which the pairs 7, 12, 14, and 17 are submetacentric, the pairs 13 and 20 are subtelocentric, and all the other pairs are metacentric (Figure 3, Table S2). *Helix lucorum* only exhibited haploid plates with *n* = 27, 10 metacentric chromosomes (1, 4, 10, 11, 14, 16, 21, 22 and 26, 27), one submetacentric (pair 2) and 16 telocentric elements (chromosome 3, 5–9, 12, 13, 15, 17–20 and 23–25) (Figure 3, Table S2).

*Campylaea planospira* (Ariantinae) and *Marmorana platychela* (Murellinae) show a karyotype of 2*n* = 60 chromosomes gradually decreasing in length; chromosomes of the two species are mostly metacentric, excluding one pair (27) of *C. planospira* and two pairs (23 and 27) of *M. platychela* that are submetacentric (Figure 3, Table S2).

### 3.3. Chromosome Banding and NOR-FISH

Chromosome banding methods were performed on samples showing an adequate number of metaphase plates, namely *Cernuella virgata*, *Cornu apertus*, *Otala lactea*, *Eobania vermiculata*, and *Theba pisana*.

**Figure 4:**
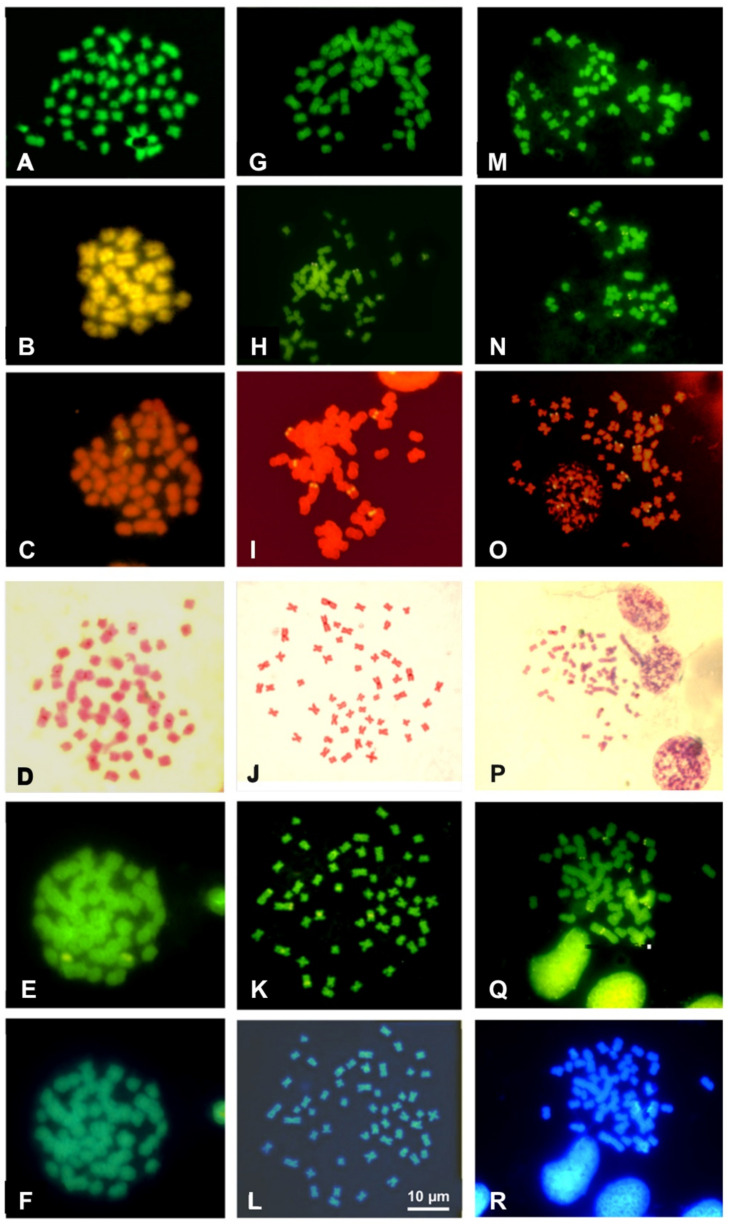
Metaphase plates of *Cernuella virgata* (**A**–**F**), *Cantareus apertus* (**G**–**L**) and *Otala lactea* (**M**–**R**) stained with Quinacrine (**A**,**G**,**M**), CMA_3_/MG (**B**,**H**,**N**), NOR-FISH (**C**,**I**,**O**), C-banding Giemsa (**D**,**J**,**P**), sequential C-banding + CMA_3_ (**E**,**K**,**Q**) + DAPI (**F**,**L**,**R**).

**Figure 5:**
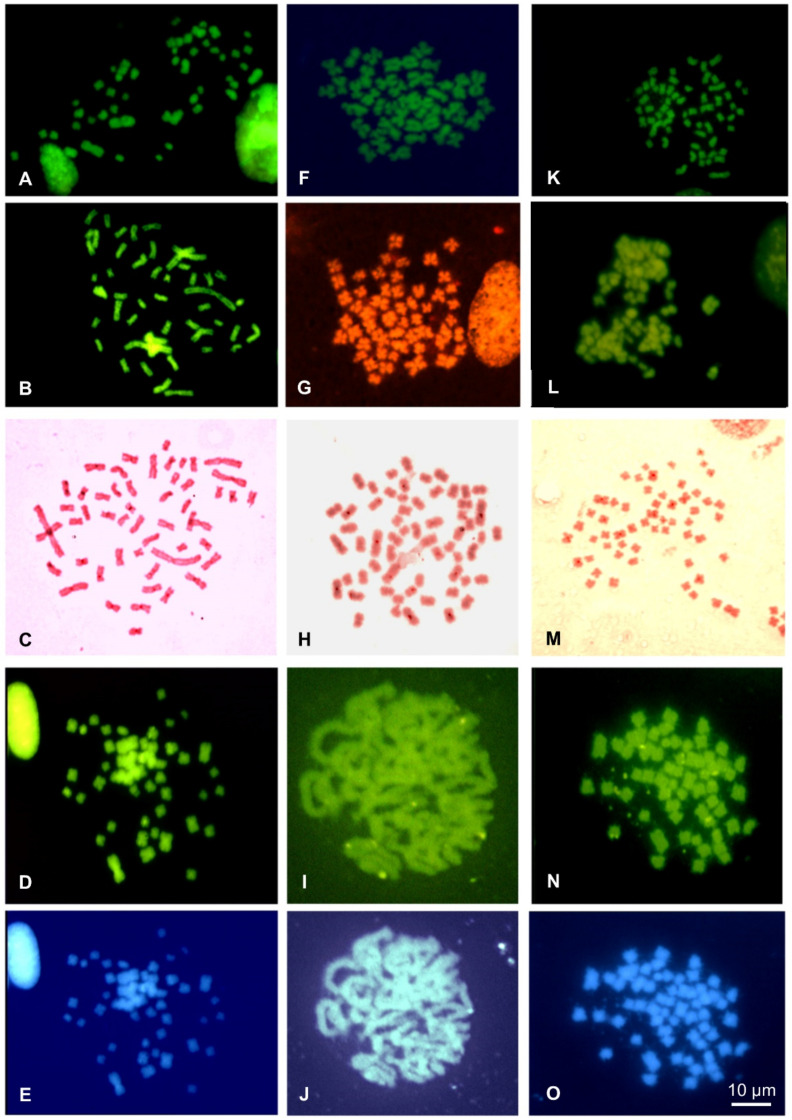
Metaphase plates of *E. vermiculata* (**A**–**E**), *Theba pisana* from Naples (**F**–**J**) and *Theba pisana* from Messina (**K**–**O**) stained with Quinacrine (**A**,**F**,**K**), CMA_3_/MG (**B**,**G**,**L**), C-banding Giemsa (**C**,**H**,**M**) and sequential C-banding + CMA_3_ (**D**,**I**,**N**) + DAPI (**E**,**J**,**O**).

Quinacrine stained uniformly all the chromosomes of all the analyzed taxa (Figure 4 and Figure 5). Staining with CMA_3_/MG evidenced two loci in *Cernuella virgata*, differentially highlighted on interstitial regions of two medium-sized chromosomes (Figure 4B). Six loci were CMA_3_/MG positive on metaphase plates of *Cornu apertus* and *Otala lactea*, two on telomeric regions of one of longest pairs (Figure 4B), while the other four loci showed an interstitial position on two pairs of medium-sized chromosomes (Figure 4H). In *Eobania vermiculata* and *Theba pisana*, CMA_3_/MG uniformly stained all chromosome pairs (Figure 5B,G,L).

Successful NOR-FISH staining was obtained on metaphase plates of *Cernuella virgata*, *Otala lactea*, and *Cornu apertus*, with hybridization signals distributed on one (*C. virgata*), three (*O. lactea*), or four pairs (*C. apertus*) of medium-sized chromosomes. CMA_3_/MG staining evidenced positive loci overlapping with those evidenced from NOR-FISH (Figure 4C,I,O). After C-banding, the five considered species showed tiny C-bands on centromeric regions of different chromosome pairs (Figure 4F,J and Figure 5C,H,M). The centromeric C-bands were Q- and DAPI negative (Figure 4E,F,K,L and Figure 5D,E,I,J,N,O), while C-banding and CMA_3_ performed on metaphase plates of *Cernuella virgata*, *Otala lactea,* and *Cornu apertus* evidenced positive loci overlapping with those highlighted with CMA_3_/MG staining and NOR-FISH. C-banding and CMA_3_ evidenced multiple pairs (2–3) showing regions positive to this fluorochromes in *Eobania vermiculata* and *Theba pisana* (Figure 4E,K and Figure 5D,I,N). The two examined populations of *T. pisana* showed very similar patterns of NORs and heterochromatin distribution (Figure 5).

## 4. Discussion

### 4.1. Molecular Analysis and Taxonomic Attribution

Searches in GenBank using the newly determined 16S rDNA sequences largely corroborated the preliminary taxonomic attribution of the study samples based on conchological and anatomical characters (Table 1). However, some considerations deserve consideration, such as the nucleotide diversification of the 16S sequences of the examined specimens of *T. pyramidata* from Capri (Naples, Italy) and the *Monacha* specimen from Portici (Naples, Italy), compared to the most similar homologous sequences deposited in GenBank. Concerning *T. pyramidata*, searches in GenBank showed an identity score of 76.3% with a specimen from Djebal Recas, (Tunisia) (AN: KY747545, [53]); 93.5% with a specimen from San Giusto, (Siena, Italy) (AN: AY741444, [54]); 92.5% with a specimen from Siena (Italy) (AN: KU521590, [55]); 93.5% with a specimen from Cala de la Mosca, Alicante (Spain) (AN: KJ458565, [9]) and 88.3% with a specimen from St. Maximin (France) (AN: AY546377, [56]). Interestingly, the 16S rRNA sequences of the two populations from Siena (Italy) show an uncorrected p-distance of 6.5%, highlighting that these populations probably require a taxonomic revision based on a comprehensive taxon sampling of their geographic distribution.

Concerning the *Monacha* specimen from Portici (Naples), it was initially attributed to *M. cartusiana* based on morphological characters, but the molecular analysis did not support this preliminary determination. In fact, the comparison of homologous 16S sequences deposited in GenBank shows that specimen of the *Monacha* here studied showed identity scores ranging from 77% to 83.5% with available specimens of *M. cartusiana*, about 85% with Russian or Lebanese species (*M. ciscaucasica*, *M. roseni*, *M. nummus*) (AN: KX495397, KX495386, KX495427, [57]), and 85.9% with the populations from Siciliy (Italy) of *Monacha* sp.1 (KX495425, [57]) (see also 16S distance matrix provided in Table S3). Furthermore, available genetic data suggest that *M. cartusiana* is genetically quite uniform, with populations from Tuscany (AN: AY741416; [54]) and Lombardy (AN: KX495378; [57]) presenting 100% identity in the 16S which, in turn, have 97% identity with Central European populations (e.g., AN: KM247391, MH204083; [58,59]). Considering the above reported molecular evidence, we here consider the specimen from Portici as a new *Monacha* candidate species, whose taxonomy and phylogenetic relationships have to be better assessed in more focused studies.

### 4.2. Chromosome Analysis and Karyotype Diversity

In this study, we performed an original molecular and chromosome analysis on different Helicoidea species and a review of all the available karyotype data on the superfamily, providing an updated taxonomic evaluation of the species so far studied (see Table S1).

We here provide for the first time karyological data on seven species of the Helicoidea superfamily: *Monaca* sp., *Trochoidea elegans*, *Trochoidea pyramidata*, *Trochoidea trochoides*, *Campylaea planospira*, *Helix gussoneana*, and *Marmorana platychela*. We also described the chromosomal formula of *Cernuella virgata*, *Helix lucorum*, *Otala lactea*, *Theba pisana* and *Cochlicella acuta*, for which only the chromosome numbers have previously been reported [25,60,61,62], (Figure 1, Figure 2 and Figure 3, Table S1). However, concerning *Cochlicella acuta*, our results disagree with the chromosome number previously provided by Aparicio [25]. In fact, the specimens examined by us had 2*n* = 52 chromosomes, while Aparicio [25] found a karyotype of 2*n* = 46 elements in specimens from Puerto de Vega (Asturias, Spain). The 16S rRNA sequence of the specimens here studied by us shows 99.1% and 97.6% identity with homologous traits of specimens of *C. acuta* from Siena and Lampedusa (Italy) (AN: AY741442 and AY741443; [54]), respectively, and 95.2% with a specimen from Bakio, Biscay (Spain) (AN: KJ458503; [9]). Unfortunately, no DNA sequences are currently available from the specimens studied in Aparicio [25]. However, considering also their very different karyotype formulae, different Mediterranean populations of *C. acuta* may belong to independent evolutionary lineages and their taxonomy should be better assessed by further molecular studies. Furthermore, our results confirm the chromosome number and morphology of *Cornu apertus*, *Erctella mazzullii*, and *Eobania vermiculata* already described in [63,64] for Sicilian specimens of these three species.

Overall, the chromosome number of the studied species ranges from 2*n* = 44 to 2*n* = 60, highlighting a significant karyological diversity in the study taxa, in line with the range from 2*n* = 42 to 2*n* = 62 so far known in Helicoidea [16] (see also Table S1). Concerning the chromosome morphology, most of the studied species have karyotypes typically containing meta- and submetacentric chromosomes, a characteristic which is commonly found in Eupulmonata [16]. However, *Helix lucorum* shows a karyotype (2*n* = 54) with 16 telocentric pairs (Figure 3). Although uncommon in Helicoidea, this karyological characteristic is not exclusive of *H. lucorum*, as karyotypes with a relatively high number of telocentric elements are showed by three Bradybaeninae, namely *Acusta ravida* (2*n* = 58), *Cathaica fasciola* (2*n* = 60), and *Bradybaena similaris* (2*n* = 56), with 7, 22 and 26 telocentric pairs, respectively [16,23,65,66] (see Table S1). Furthermore, in Alopiinae, *Medora* sp. shows a karyotype (2*n* = 62) with 11 telocentric pairs [31].

**Figure 6:**
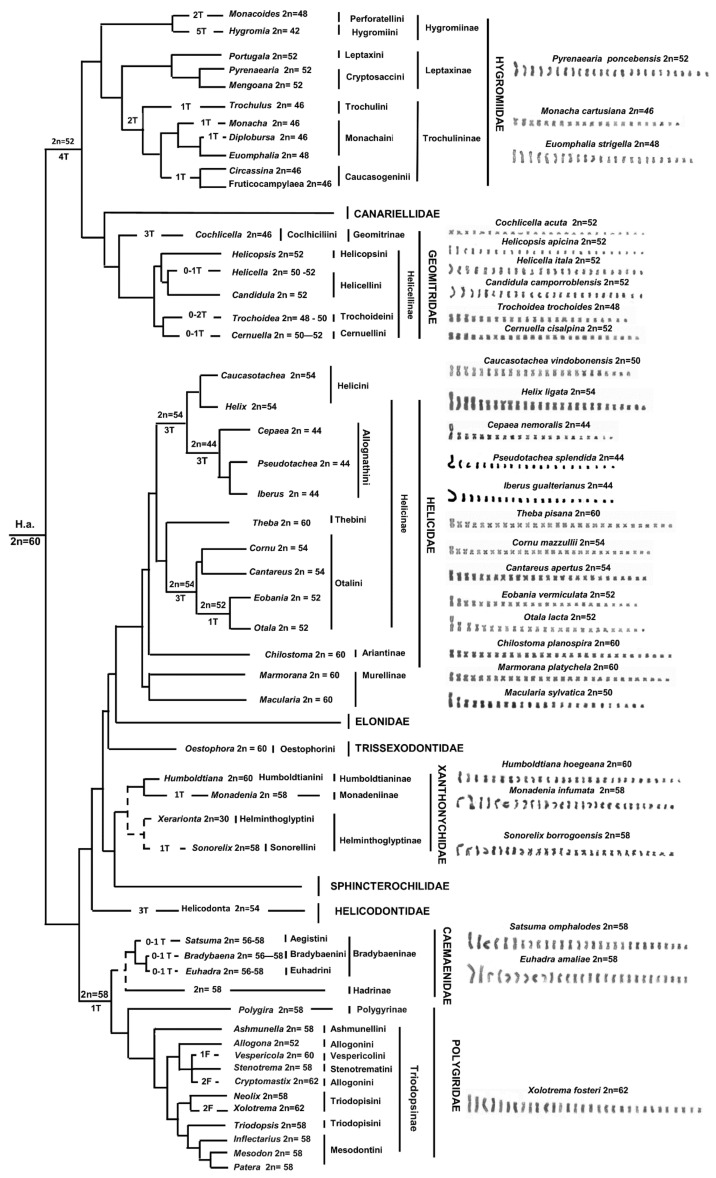
Phylogenetic tree of Helicoidea (redrawn from [9]) superimposed with our hypothesis on chromosome diversification in Helicoidea. Evolutionary relationships of Hygromiidae, Polygiridae, and Ariantine are from [13,14]. T = translocation; F = Fission. Dashed lines = uncertain relationships. Haploid karyotypes were redrawn from figures presented in the original papers (for References see Table S1; in particular, for *Cepaea nemoralis*, for which two formulas are given, the presented haploid karyotype is from [24]).

Variations in chromosome number and morphology in the Helicoidea superfamily, and more in general in Eupulmonata, are considered taxonomically relevant and have been highly debated in past studies, with some authors suggesting a progressive reduction of the chromosome number [9,67], while others supporting the opposite hypothesis [16,60]. In this regard, to evaluate the evolutionary trends of karyotype variations in the studied taxa, we superimposed on the evolutionary relationships of the Helicoidea of the Western Palearctic [9,10,14] all the available chromosome data as listed in Table S1, with an updated nomenclature following WoRMS [1] and MolluscaBase [2] (Figure 6).

In our hypothesis, accounting for both chromosome number and morphology, we considered a karyotype composed of 2*n* = 60 as the putative ancestral condition in Helicoidea (Figure 6). This assumption is based on two main considerations: (i) this karyotype is conserved in different families and subfamilies without any noticeable modification; (ii) the most parsimonious hypothesis on chromosomal diversification in the superfamily (with a lower number of chromosome rearrangement per lineage) should account for an overall reduction of the chromosome number from 2*n* = 60 to 2*n* = 42. This probably occurred by means of multiple independent, tandem fusions/translocation in different evolutionary lineages. Furthermore, while the putative ancestral karyotype of 2*n* = 60 shows a conserved morphology in the Helicoidea phylogeny, with mostly metacentric elements gradually decreasing in length (e.g., *Marmorana platychela* and *Theba pisana*, present study), distinctively larger pairs are clearly visible in karyotypes with a relatively low chromosome count (2*n* = 44–42) (e.g., *Cepaea* or *Iberus* species, reference in [16], as a clear result of a progressive accumulation of translocations). On the other hand, a general tendency toward an overall decrease in the chromosome number has been hypothesized also in other Gastropoda (e.g., Opisthobranchia and Cephalaspidea [16]), thus possibly representing a significant chromosomal evolutionary trajectory of several groups.

In particular, in Helicoidea, the karyotype of the common ancestor of the clade, including Hygromiidae and Geomitridae (2*n* = 52), was likely shaped by four chromosome translocations. In the former family, the Leptaxinae inherited this ancestral condition, while in the Hygromiinae, two and five translocations would have produced the karyotype of 2*n* = 48 in Perforatellini and 2*n* = 42 in Hygromiini, respectively. In Trochulininae, most species have a karyotype of 2*n* = 46, which probably originated from the ancestral 2*n* = 52 by means of three translocations. In Geomitridae, most species of the different subfamilies and tribes show a conserved karyotype of 2*n* = 52, except for Trochodeini, which shows karyotypes of 2*n* = 50 (*Xerograssa*) and 2*n* = 48 (*Trochoidea*), which probably originated from one and two translocations, respectively.

The putative primitive Helicoidea karyotype of 2*n* = 60 is conserved in Trissexodontidae, Xanthonychidae, and some taxa of Helicidae, namely tribes Ariantinae and Thebini, the Murellinae *Marmorana platychela* and the Helicini *Caucasotachea leucoranea*. Several species of Otalini and most Helicini have 2*n* = 52–54, while all Allognathini have 2*n* = 44, so their karyotype could have been originated from the ancestral 2*n* = 60 condition by means of a progressive accumulation (three to eight) of translocations. Notably, in Helicidae, the species of several tribes of Helicinae have the chromosomes of pair 1 distinctively larger than the elements of pair 2, (Figure 2, Figure 3 and Figure 6; Table S2), suggesting that the pair 1 was a preferential site for translocations occurred during the transition from 2*n* = 60 to 2*n* = 44. This condition is present also in *Macularia sylvatica* (Murellinae) (see also [16]), suggesting that similar processes occurred independently in different taxonomic groups.

In Helicontidae, the karyotype of 2*n* = 54 of *Helicodonta obvoluta* originated from three translocations, while in the clade including Polygeridae and Camaenidae, a single translocation originated the karyotype of 2*n* = 58 of their common ancestor, which is conserved in most of the 80 Camaenidae and the about 50 Polygeridae species so far analysed. The few exceptions are represented by some Bradybaeninae, whose karyotype of 2*n* = 56 likely originated by means of one translocation event, while the karyotype of 2*n* = 60 of *Cathaica fasciola* probably originated from one fission. Among Polygyridae, the putative ancestral karyotype of the family (2*n* = 58) is conserved in most studied species, and deviations from this condition concern either a reduction (2*n* = 52, two *Allogona* species) or an increase in the chromosome number (2*n* = 60 in *Vespericola columbiana*, and 2*n* = 62 in *Cryptomastix germana*, *Xolotrema fosteri* and *Triodopsis fraudolenta*), involving a progressive accumulation of chromosome translocations and fissions, respectively.

Besides translocation and rare fission events, the available data suggest that also chromosome inversions were relevant to chromosome rearrangements in the karyotype diversification of the Helicoidea. In fact, a progressive accumulation of chromosome inversions explains the differences in the overall karyotype morphology exhibited in different Bradybaeninae genera (e.g., *Acusta* and *Fruticicola*, [16]), *Trochoidea* (2*n* = 48) and *Helix* (2*n* = 54) (present study). In *Trochoidea*, three and four inversions occurred from the karyotype of *T. pyramidata* (20 m, 4 sm) to those of *T. trochoides* (16 m, 7 sm, 1 t) and *T. elegans* (16 m, 6 sm, 2 t), respectively (Figure 1; Table S1). In *Helix*, a progressive accumulation of three, six, and sixteen inversions likely occurred from the karyotype of *H*. *straminea* (24 m, 2 sm, 1st) [68] to those of *H. gussoneana* (21 m, 4 sm, 2st), *H. pomatia* (18 m, 8 sm, 1st), and *H. lucorum* (10 m, 1 sm, 16 t), respectively (Figure 3; Table S1).

Loci of NORs are generally considered useful taxonomic and phylogenetic markers [69,70,71]. Their localization on a single chromosome pair is considered a primitive character in molluscs, while their occurrence on multiple pairs is regarded as a derived state [16,30,70,72,73,74,75]. Both conditions are present in Helicoidea, but the available data are still scarce to draw phylogenetic considerations. Loci of NORs are on a single pair in two Geometridae species (*Helicella virgata*, [76]; *Cernuella cisalpina*, this study) and the polygyrid *Xolotrema fosteri* [77] and on multiple chromosome pairs in Helicidae (five species of Otalini and *Theba pisana*; [64], this study) (Table S1). Similarly, studies concerning the location and composition of heterochromatin in Helicoidea concern only one species of Geomitridae and six Helicidae species (see also [63,64]) (Table S1). In these species, tiny heterochromatic C-bands are prevalently localized on centromeric and pericentromeric regions, resulting in Q and DAPI being negative and suggesting a very limited presence of A-T rich clusters [78]. In contrast, C-banding and CMA_3_ highlighted NOR-associated heterochromatin, which is notoriously rich in G-C [30,79,80,81].

## 5. Conclusions

We here provide new molecular and cytogenetic data on 15 Helicoidea (Eupulmonata) species and a synthesis on all the available karyological data on the superfamily. The newly generated cytogenetic data include four Hygromiidae, two Geometridae, and nine Helicidae, which show a significant chromosome diversity with karyotypes ranging from 2*n* = 44 to 2*n* = 60. Considering the available karyological and phylogenetic data, we hypothesize a karyotype of 2*n* = 60 with all biarmed elements gradually deceasing in length as the ancestral condition in the superfamily Helicoidea. A reduction of the chromosome number, by means of a progressive accumulation of chromosome translocations, led to the formation of karyotypes with a lower chromosome number (to 2*n* = 50–44). This process occurred multiple times and independently among different evolutionary lineages, while the opposite process, an augment of the total chromosome count by means of chromosome fissions, is detectable in Polygyridae. Other than translocations and rare fissions, chromosome inversions were relevant to chromosome rearrangements in Helicoidea, leading to the formation of telocentric elements in karyotypes with a relatively low chromosome count.

## Figures and Tables

**Table 1:** Number and provenance of the examined specimens of Helicoidae. Biological samples (methanol and acetic acid fixed cell suspensions) are deposited in the Molluscan collection of the Department of Biology, University of Naples Federico II. * Present study; Chr. Nr = chromosome number.

Family/Subfam/Tribe	Species	Nr. and Origin of Samples	Voucher	Chr. Nr.
**Hygromiidae**				
**Hygromiinae**				
Monachaini	*Monacha* sp.	1, Portici (Naples, Italy)	Gast 32 85.9% vs. KX495397	2*n* = 44 *
Trochoideini	*Trochoidea elegans* (Gmelin, 1791)	3, Santa Severa (Rome, Italy)	Gast 193–195 99.3% vs. MG585435	2*n* = 48 *
	*Trochoidea pyramidata* (Draparnaud, 1805)	3, Capri (Naples, Italy)	Gast 184–186 93.9% vs. AY741444	2*n* = 48 *
	*Trochoidea trochoides* (Poiret, 1789)	2, Fusaro (Naples, Italy)	Gast 91, 170 98.3% vs. AY546379	2*n* = 48 *
**Geomitridae**				
**Helicellinae**				
Cernuellini	*Cernuella virgata* (Da Costa, 1778)	2, Seiano (Naples, Italy)	Gast 354, 355 100% vs. KF250441	2*n* = 52
Cochlicellini	*Cochlicella acuta* (O. F. Müller, 1774)	2, Monte S.’Angelo (Naples, Italy)	Gast 342–343 100% vs. AY741443	2*n* = 52
**Helicidae**				
**Ariantinae**	*Campylaea planospira* (Lamarck, 1822)	2, Amalfi (Salerno, Italy)	Gast 202–203	2*n* = 60 *
**Helicinae**				
Helicini	*Helix gussoneana* L. Pfeiffer, 1848	1, Petina (Salerno, Italy)	Gast 149 99.7% vs. KU869969	2*n* = 54 *
	*Helix lucorum* Linnaeus, 1758	2, Montellago (Venice, Italy)	Gast 352–353 99.3% vs. MG709101	2*n* = 54
Otalini	*Cornu apertus* (Born, 1778)	2, Frignano (Caserta, Italy)	Gast 357–358 97.7% vs. KU870010	2*n* = 54
	*Eobania vermiculata* (O. F. Müller, 1774)	1, Capri (Naples, Italy)	Gast 356	2*n* = 52
	*Erctella mazzullii* (De Cristofori & Jan, 1832)	1, Palermo (Italy)	Gast 67 99.5% vs. GQ402415	2*n* = 54
	*Otala lactea* (O. F. Müller, 1774)	2, Morocco	Gast 23–24 100% vs. MK603015	2*n* = 52
Thebini	*Theba pisana* (O. F. Müller, 1774)	2, Fusaro (Naples, Italy)	Gast 87, 172 98.3% vs. AY741415	2*n* = 60
	*Theba pisana* (O. F. Müller, 1774)	1, Messina (Italy)	Gast 77 99.2% vs. KU521652	2*n* = 60
**Murellinae**	*Marmorana platychela* (Menke, 1830)	2, Palermo (Italy)	Gast 66, 108 100% vs. MG774447	2*n* = 60 *

## Data Availability

The data presented in this study are available in the manuscript and in Tables S1–S3. All the newly generated DNA sequences were submitted to GenBank (accession numbers: MZ504244-MZ504269).

## Supplementary Materials

The following are available online at www.mdpi.com/article/10.3390/ani11092551/s1. Table S1. Available karyological data on Helicoidea; Table S2. Relative length (RL) and Centromeric Index (CI) of the studied taxa. Table S3. Distance Matrix of the Monacha species considered in the study.
