# Supplementary material for: Chromosome Diversity and Evolution in Helicoide a (Gastropoda: Stylommatophora): A Synthesis from Original and Literature Data
Source: Animals (Basel). 2021 Aug 30;11(9):2551. doi: 10.3390/ani11092551 (PMC8470273; doi:10.3390/ani11092551)
Supplement: Supplementary file 1 [file animals-11-02551-s001.zip › animals-1326230-supplementary.pdf]

**Table S1**. List of available karyological data on Helicoidea. Taxonomy and nomenclature follow WoRMS (2021) [1] and MolluscaBase (2021) [2]. m = metacentric, sm = submetacentric, st = subtelocentric, t = telocentric; n e 2n = haploid and diploid chromosome number; ? = uncertain taxonomic attribution. C = C-banding; NOR = Ag-NOR staining; FISH = Fluorescence in situ hybridization.

| **Family**<br>**Subfamily** | **Tribe**<br>**Species** | **Synonym used in the original paper** | **Origin** | **n** | **2n** | **Karyotype** | **Method** | **Reference** |
|---|---|---|---|---|---|---|---|---|
| **Camaenidae** | | | | | | | | |
| **Bradybaeninae** | **Bradybaenini** | | | | | | | |
| | *Acusta despecta* (G.B. Sowerby I, 1839) | *Fruticicola despecta* | Japan | 29 | 58 | | | [in 3] |
| | *Acusta despecta sieboldtiana* (Pfeiffer, 1850) | | Korea | 29 | 58 | 12m, 17sm | | [in 4] |
| | *Acusta despecta sieboldtiana* (Pfeiffer, 1850) | | Japan | 29 | 58 | 21m, 7sm, 1st | | [in 4] |
| | *Acusta ravida* (Benson, 1842) | *Bradybaena ravida* | China | 29 | 58 | 19m, 3sm, 7t | | [in 4] |
| | *Bradybaena similaris* (Férussac, 1822) | | Japan | 28 | 56 | | | [in 3] |
| | *Bradybaena similaris* (Férussac, 1822) | | Japan | 28 | 56 | 2m, 2/3sm, 24/23t | | [5] |
| | *Cathaica fasciola* (Draparnaud, 1801) | | China | 30 | 60 | 7m, 1sm, 22t | | [in 4] |
| | *Ezohelix gainesi* (Pilsbry, 1900) | *Bradybaena gainesi* | Japan | 28 | 56 | | | [in 3] |
| | *Ezohelix gainesi* (Pilsbry, 1900) | *Bradybaena gainesi* | Korea | 29 | 58 | | | [in 6] |
| | *Fruticicola fruticum* (O.F. Müller, 1774) | *Eulota fruticum* | Europa | 29 | 58 | | | [in 3] |
| | *Fruticicola koreana* (Pfeiffer, 1850) | *Koreanohadra koreana* | Korea | 29 | 58 | 23m, 5sm, 1t | | [in 6] |
| | *Fruticicola koreana* (Pfeiffer, 1850) | *Koreanohadra koreana* | Korea | 29 | 58 | 10m, 13sm, 1st, 5t | | [in 4] |
| | *Fruticicola koreana* (Pfeiffer, 1850) | *Koreanohadra koreana* | Korea | 29 | 58 | 16m, 12sm,1st | | [6] |
| | *Fruticicola kurodana* (Pilsbry, 1926) | *Koreanohadra kurodana* | Korea | 29 | 58 | 11m, 17sm, 1t | | [in 4] |
| | *Karaftohelix adamsi* (Kuroda & Hukuda, 1944) | *Koreanohadra adamsi* | Korea | 29 | 58 | 10m, 18sm, 1st | | [in 4] |
| | **Aegistini** | | | | | | | |
| | *Aegista chosenica* (Pilsbry, 1927) | | Korea | 28 | 56 | 10m, 18sm | | [in 4] |
| | *Aegista diversa* Kuroda & Miyanaga, 1936 | | Korea | 28 | 56 | 14m, 14sm | | [in 4] |
| | *Aegista kiusiuensis* (Pilsbry, 1900) | *Aegista vatheleti* | Japan | 29 | 58 | | | [in 3] |
| | *Aegista quelpartensis* (Pilsbry & Hirase, 1908) | | Korea | 28 | 56 | 15m, 10sm, 3st | | [in 4] |
| | *Nesiohelix samarangae* (Kuroda & Miyanaga, 1943) | | Korea | 29 | 58 | 12m, 9sm, 2st, 1t | | [in 4] |
| | *Satsuma japonica* (L. Pfeiffer, 1847) | | Japan | 29 | 58 | 19m, 10sm | | [7] |
| | *Satsuma myomphala* (Martens, 1865) | | Japan | 29 | 58 | | | [in 3] |
| | *Satsuma omphalodes* (Pilsbry, 1901) | | Japan | 29 | 58 | 19m, 10sm | | [in 4] |
| | **Euhadrini** | | | | | | | |
| | *Euhadra amaliae* (Kobelt, 1875) | | Japan | 28 | 56 | 19m, 9sm | | [in 4] |
| | *Euhadra awaensis* (Pilsbry, 1902) | | Japan | 29 | 58 | | | [in 3] |
| | *Euhadra callizona* (Crosse, 1871) | | Japan | 28 | 56 | | | [in 3] |
| | *Euhadra callizon*a (Crosse, 1871) | | Japan | 28 | 56 | 19m, 9sm | | [in 4] |
| | *Euhadra congenita* (E.A. Smith, 1878) | | Japan | 28 | 56 | | | [in 3] |
| | *Euhadra dixoni* (Pilsbry, 1900) | | Japan | 28 | 56 | 19m, 9sm | | [in 4] |

| | | | | | | | |
|---|---|---|---|---|---|---|---|
| | *Euhadra eoa* (Crosse, 1868) | | Japan | 28 | 56 | | [in 3] |
| | *Euhadra eoa* (Crosse, 1868) | | Japan | 28 | 56 | 19m, 9sm | [in 4] |
| | *Euhadra grata* (Gude, 1900) | | Japan | 28 | 56 | | [in 3] |
| | *Euhadra idzumonis* (Pilsbry & Gulick, 1900) | | Japan | 29 | 58 | | [in 3] |
| | *Euhadra peliomphala* (L. Pfeiffer, 1850) | | Japan | 28 | 56 | | [in 3] |
| | *Euhadra peliomphala simodae* (Jay, 1856) | | Japan | 28 | 56 | 18m, 10sm | [in 4] |
| | *Euhadra quaesita* (Deshayes, 1850) | | Japan | 29 | 56 | | [in 3] |
| | *Euhadra sandai* (Kobelt, 1879) | | Japan | 28 | 56 | | [in 6] |
| | *Euhadra sandai oki* Pilsbry, 1928 | | Japan | 28 | 56 | 21m, 7sm | [in 4] |
| | *Euhadra sandoensis* (Pilsbry & Y. Hirase, 1903) | | Japan | 28 | 56 | | [in 3] |
| | *Euhadra scaevola* (Martens, 1877) | | Japan | 29 | 58 | | [in 3] |
| | *Euhadra senckenbergiana* (Kobelt, 1875) | | Japan | 28 | 56 | | [in 3] |
| | *Euhadra senckenbergiana* (Kobelt, 1875) | | Japan | 28 | 56 | | [in 6] |
| | *Euhadra subnimbosa* (Kobelt, 1894) | | Japan | 28 | 56 | 21m, 9sm | [in 4] |
| **Hadrinae** | | | | | | | |
| | *Bentosites macleayi* (J.C. Cox, 1865) | | Australia | 29 | 58 | | [8] |
| | *Catellotrachia setigera* (Tate, 1894) | *Semotrachia setigera* | Australia | 29 | 58 | | [8] |
| | *Chloritobadistes sp*. Iredale, 1933 | | Australia | 29 | 58 | | [8] |
| | *Chloritobadistes victoriae* (Cox, 1868) | | Australia | 29 | 58 | | [8] |
| | *Cupedora cassandra* (Pfeiffer, 1864) | *Meracomelon cassandra* | Australia | 29 | 58 | | [8] |
| | *Cupedora evandaleana* (Pfeiffer, 1864) | | Australia | 29 | 58 | | [8] |
| | *Cupedora patruelis* (Angas, 1864) | | Australia | 29 | 58 | | [8] |
| | *Cupedora rufofasciata* Brazier, 1875 | *Meracomelon rufofasciatum* | Australia | 29 | 58 | | [8] |
| | *Cupedora* sp. Iredale, 1937 | | Australia | 29 | 58 | | [in 3] |
| | *Cupedora* sp. Iredale, 1937 | | Australia | 29 | 58 | | [8] |
| | *Cupedora sutilosa* (Férussac, 1839) | *Exilibadistes sutilosa* | Australia | 29 | 58 | | [8] |
| | *Glyptorhagada kooringensis* (Angas, 1877) | | Australia | 29 | 58 | | [8] |
| | *Granulomelon adcockianum* (Bednall, 1894) | *Pleuoxia adcockiana* | Australia | 29 | 58 | | [8] |
| | *Hadra* sp. E.von Martens, 1860 | | Australia | 29 | 58 | | [in 3] |
| | *Meracomelon moorundianum* T. Iredale, 1937 | | Australia | 29 | 58 | | [8] |
| | *Meracamelon sp.* | | Australia | 29 | 58 | | [in 3] |
| | *Meracomelon subloriolianum* (Pilsbry,1890) | | Australia | 29 | 58 | | [8] |
| | *Papuina* sp. E. von Martens, 1860 | | Australia | 29 | 58 | | [in 3] |
| | *Parglogenia pelodes* (Pfeiffer, 1846) | | Australia | 29 | 58 | | [8] |
| | *Planispira fallaciosa* (A.E.J. Férussac, 1821) | | Australia | 29 | 58 | | [in 3] |
| | *Pleuroxia* sp. Ancey, 1887 | | Australia | 29 | 58 | | [in 3] |
| | *Pleuroxia* sp. Ancey, 1887 | | Australia | 29 | 58 | | [8] |

| | | | | | | | | | |
|---|---|---|---|---|---|---|---|---|---|
| | | *Pommerhelix depressa* (Hedley, 1901) n. comb. | *Meridiolum depressum* | Australia | 29 | 58 | | | [8] |
| | | *Sinumelon bednalli* (Ponsonby, 1904) | | Australia | 29 | 58 | | | [8] |
| | | *Sinumelon flindersi* (Adams & Angas, 1864) | | Australia | 29 | 58 | | | [8] |
| | | *Sinumelon nullaboricum* (Tate, 1879) | | Australia | 29 | 58 | | | [8] |
| | | *Sinumelon perinflatum* (Pfeiffer, 1864) | | Australia | 29 | 58 | | | [8] |
| | | *Sinumelon remissum* Iredale, 1937 | | Australia | 29 | 58 | | | [8] |
| | | *Sinumelon* sp. Iredale, 1937 | | Australia | 29 | 58 | | | [in 3] |
| | | *Sphaerospira fraseri* (Gray in Griffith & Pidgeon, | | Australia | 29 | 58 | | | [8] |
| | | *Strepsitaurus rugus* (Cotton, 1953) | *Pleuroxia ruga* | Australia | 29 | 58 | | | [8] |
| | | *Torresitrachia stipata* Iredale, 1938 | | Australia | 29 | 58 | | | [8] |
| | | *Xanthomelon durvillii* (Hombron & Jacquinot, 1841) | | Australia | 29 | 58 | | | [8] |
| | | *Xanthomelon* sp. E. von Martens, 1860 | | Australia | 29 | 58 | | | [8] |
| **Geomitridae** | | | | | | | | | |
| | **Helicellinae** | **Cernuellini** | | | | | | | |
| | | *Cernuella neglecta* (Draparnaud, 1805) | | Australia | 26 | 52 | | | [in 3] |
| | | *Cernuella virgata* (Da Costa, 1778) | *Helicella virgata* | Australia | 26 | 52 | | | [in 3] |
| | | *Cernuella virgata* (Da Costa, 1778) | | Spain | 26 | 52 | | | [9] |
| | | *Cernuella virgata* (Da Costa, 1778) | | Italy | 26 | 52 | 24m, 2sm | C, NOR | [10]; this study |
| | | *Xerosecta cespitum* (Draparnaud, 1801) | *Cernuella cespitum* | Spain | 25 | 50 | | | [9] |
| | | *Xerosecta reboudiana* (Bourguignat, 1863) | *Cernuella reboudiana* | Spain | 25 | 50 | | | [9] |
| | | **Cochlicellini** | | | | | | | |
| | | *Cochlicella acuta* (O.F. Müller, 1774) | | Europe | 23 | 46 | | | [in 3] |
| | | *Cochlicella acuta* (O.F. Müller, 1774) | | Spain | 23 | 46 | | | [9] |
| | | *Cochlicella acuta* (O.F. Müller, 1774) | | Italy | 26 | 52 | 18m, 5sm, 2st, 1t | | this study |
| | | *Cochlicella barbara* (Linnaeus, 1758) | *Cochlicella ventrosa* | Australia | 23 | 46 | | | [in 3] |
| | | **Helicellini** | | | | | | | |
| | | *Candidula camporroblensis* (Fez, 1944) | | Spain | 26 | 52 | | | [9] |
| | | *Candidula unifasciata* (Poiret, 1801) | | Europe | 27 | 52 | | | [in 3] |
| | | *Helicella gigaxi* (O.F. Müller, 1774) | *Candidula gigaxi* | Spain | 26 | 52 | | | [9] |
| | | *Helicella itala* (Linnaeus, 1758) | | Spain | 26 | 52 | | | [9] |
| | | *Helicella itala* (Linnaeus, 1758) | | Europe | 26 | 52 | | | [in 3] |
| | | *Helicella ordunensis* (Kobelt, 1883) | | Spain | 26 | 52 | | | [in 3] |
| | | *Helicella stiparum* (Rossmässler, 1854) | | Spain | 25 | 50 | | | [in 3] |
| | | *Microxeromagna lowei* (Potiez & Michaud, 1838) | *Helicella virgata* | Australia | 26 | 52 | | | [in 3] |
| | | *Xerolenta obvia* (Menke, 1828) | Helicella obvia | Spain | 26 | 52 | | | [in 3] |
| | | *Xeroplexa intersecta* (Poiret, 1801) | *Candidula intersecta* | Spain | 26 | 52 | | | [11] |
| | | *Xerotricha apicina* (Lamarck, 1822) | *Helicopsis apicina* | Portugal | 26 | 52 | 23m, 2sm | | [in 4] |

| | | | | | | |
|---|---|---|---|---|---|---|
| *Zarateana rocandioi* (Ortiz de Zárate y López, 1950) | *Candidula rocandioi* | Portugal | 26 | 52 | | [9] |
| **Helicopsini** | | | | | | |
| *Xeropicta derbentina* (Krynicki, 1836) | | Georgia | 26 | 52 | | [12] |
| **Trochoideini** | | | | | | |
| *Trochoidea elegans* (Gmelin, 1791) | | Italy | 24 | 48 | 16m, 6sm, 2t | this study |
| *Trochoidea pyramidata* (J.P.R. Draparnaud, 1805) | | Italy | 24 | 48 | 20m, 4sm | this study |
| *Trochoidea trochoides* (Poiret, 1789) | | Italy | 24 | 48 | 16m, 7sm, 1t | this study |
| *Xerocrassa cretica* (L. Pfeiffer, 1841) | | Turkey | 26 | 52 | | [in 3] |
| *Xerocrassa geyeri* (Soós, 1926) | | Spain | 25 | 50 | | [9] |
| **Helicidae** | | | | | | |
| **Ariantinae** | | | | | | |
| *Arianta arbustorum* (Linnaeus, 1758) | *Helicigona arbustorum* | Europe | 30 | 60 | | [in 3] |
| *Campylaea planospira* (Lamarck, 1822) | | Italy | 30 | 60 | 29m, 1sm | this study |
| *Causa holosericea* (S. Studer, 1820) | *Isognomostoma holosericum* | Europe | 30 | 60 | | [in 3] |
| *Chilostoma achates aethica* (Rossmässler, 1835) | *Helicigona achates aethica* | Europe | 30 | 60 | | [in 3] |
| *Helicigona lapicida* (Linnaeus, 1758) | | Europe | 29 | 58 | | [in 3] |
| *Helicigona pouzolzii* (Deshayes, 1832) | | Europe | 29 | 58 | | [in 3] |
| *Helicigona setosa* (Férussac, 1832) | | Europe | 29 | 58 | | [in 3] |
| *Isognomostoma isognomostomos* (Schröter, 1784) | | Europe | 30 | 60 | | [in 3] |
| **Helicinae** | | | | | | |
| **Allognathini** | | | | | | |
| *Cepaea hortensis* (O.F. Müller, 1774) | | UK | 22 | 44 | m/sm/st | [in 4] |
| *Cepaea nemoralis* (Linnæus, 1758) | | Europe | 22 | 44 | | [in 3] |
| *Cepaea nemoralis* (Linnæus, 1758) | | UK | 22 | 44 | 13m, 9sm | [13] |
| *Cepaea nemoralis (*Linnæus, 1758) | | UK | 22 | 44 | 14m, 8sm | [14] |
| *Cepaea nemoralis* (Linnæus, 1758) | | UK | 22 | 44 | m/sm/st | [in 4] |
| *Iberus alonensis* (Férussac,1821) | | Spain | 22 | 44 | m/sm | [in 4] |
| *Iberus gualterianus* (Linnaeus, 1758) | | Spain | 22 | 44 | m/sm | [in 4] |
| *Iberus guiraonus* Rossmäessler in Pfeiffer, 1853)? | | Spain | 22 | 44 | m/sm | [in 4] |
| *Iberus marmoratus* (Férussac, 1821) | | Spain | 22 | 44 | m/sm | [in 4] |
| *Pseudotachea liturata* (L. Pfeiffer, 1851) | | Europe | 22 | 44 | | [in 3] |
| *Pseudotachea liturata* (L. Pfeiffer, 1851) | | Spain | 22 | 44 | | [11] |
| *Pseudotachea splendida* (Draparnaud, 1801) | | Spain | 22 | 44 | m/sm | [in 4] |
| **Helicini** | | | | | | |
| *Caucasotachea atrolabiata calligera* (Dubois de | | Georgia | 26 | 52 | | [15] |
| *Caucasotachea calligera* (Dubois de Montpéreux, | | Georgia | 26 | 52 | | [15] |
| *Caucasotachea leucoranea* (Mousson, 1863) | *Caucasotachea leukoranea* | Iran | 30 | 60 | | [in 3] |

| | | | | | | | | |
|---|---|---|---|---|---|---|---|---|
| | *Caucasotachea vindobonensis* (C. Pfeiffer, 1828) | *Cepea vindobonensis* | UK | 25 | 50 | m/sm/st | | [in 4] |
| | *Helix buchi* Dubois de Montpéreux, 1839 | | Geogia | 27 | 54 | | | [15] |
| | *Helix cincta* (O.F. Müller, 1774) | | Europe | 27 | 54 | | | [in 3] |
| | *Helix dormitoris bosnica* W. Kobelt, 1898 | | Europe | 27 | 54 | | | [in 3] |
| | *Helix gussoneana* L. Pfeiffer, 1848 | | Italy | 27 | 54 | 21m, 4sm, 2st | | this study |
| | *Helix lucorum* Linnaeus, 1758 | | Georgia | 27 | 54 | | | [15] |
| | *Helix lucorum* Linnaeus, 1758 | | Italy | 27 | 54 | 10m, 1sm, 16t | | this study |
| | *Helix lucorum trapezuntis* (Forcart, 1963) | | Europe | 27 | 54 | | | [in 3] |
| | *Helix melanostoma* Draparnaud, 1801 | | Europe | 27 | 54 | | | [in 3] |
| | *Helix pomatia* (Linnaeus, 1758) | | Europe | 27 | 54 | 18m, 8sm, 1st | | [in 3] |
| | *Helix straminea* Briganti, 1825 | | Italy | 27 | 54 | 24m, 2sm, 1st | | [16] |
| | **Otalini** | | | | | | | |
| | *Cornu apertus* (Born, 1778) | *Helix aperta* | Europe | 27 | 54 | | | [in 3] |
| | *Cornu apertus* (Born, 1778) | *Helix aperta* | Italy | 27 | 54 | 22m, 4sm, 1st | NOR, C, FISH | [17]; this study |
| | *Cornu aspersum* (O.F. Müller, 1774) | *Helix aspersa* | Europe | 27 | 54 | | | [in 3] |
| | *Cornu aspersum* (O.F. Müller, 1774) | *Cantareus aspersus* | Italy | 27 | 54 | 27m | NOR, C, FISH | [17] |
| | *Eobania vermiculata* (O.F. Müller, 1774) | | Spain | 26 | 52 | mostly m | | [9, 18] |
| | *Eobania vermiculata* (O.F. Müller, 1774) | | Italy | 26 | 52 | 24m, 2sm | NOR, C, FISH | [17]; this study |
| | *Eobania vermiculata* (O.F. Müller, 1774) | | Europe | 26 | 52 | | | [in 3] |
| | *Erctella mazzullii* (De Cristofori & Jan, 1832) | *Cantareus mazzullii* | Italy | 27 | 54 | 25m, 2sm | NOR, C, FISH | [17]; this study |
| | *Loxana alabastrites* (Michaud, 1833) | *Iberus soluta* | Europe | 25 | 50 | | | [in 3] |
| | *Otala dupotetiana* (A. Terver, 1839) | *Archelix dupotetiana* | Europe | 26 | 52 | | | [in 3] |
| | *Otala hieroglyphicula* (Michaud, 1833) | Archelix *hieroglyphicula* | Europe | 26 | 52 | | | [in 3] |
| | *Otala lactea* (O.F. Müller, 1774) | | Marocco | 26 | 52 | 20m, 5sm, 1t | NOR, C, FISH | this study |
| | *Otala lactea* (O.F. Müller, 1774) | | Spain | 26 | 52 | | | [in 4] |
| | *Otala punctata* (O.F. Müller, 1774) | *Archelix punctata* | Europe | 26 | 52 | | | [in 3] |
| | *Otala xanthodon* (Anton, 1839) | *Archelix xanthodon* | Europe | 26 | 52 | | | [in 3] |
| | **Thebini** | | | | | | | [in 3] |
| | *Theba pisana* (O.F. Müller, 1774) | *Euparypha pisana* | Australia | 30 | 60 | | | [in 3] |
| | *Theba pisana* (O.F. Müller, 1774) | *Euparypha pisana* | Europe | 28 | 56 | | | [in 3] |
| | *Theba pisana* (O.F. Müller, 1774) | | Spain | 30 | 60 | | | [18] |
| | *Theba pisana* (O.F. Müller, 1774) | | Italy | 30 | 60 | 30m | NOR, C, FISH | this study |
| **Murellinae** | | | | | | | | |
| | *Macularia sylvatica* (Draparnaud, 1801) | *Cepea silvatica* | Europe | 25 | 50 | | | [in 3] |
| | *Macularia sylvatica* (Draparnaud, 1801) | *Cepea sylvatica* | UK | 25 | 50 | m/sm/st | | [in 4] |
| | *Marmorana platychela* (Menke, 1830) | | Italy | 30 | 60 | 28m, 2sm | | this study |
| **Helicodontidae** | | | | | | | | |

| | | | | | | | | | |
|---|---|---|---|---|---|---|---|---|---|
| | **Helicondontinae** | | | | | | | | |
| | | | *Helicodonta obvoluta* (O.F. Müller, 1774) | | Europe | 27 | 54 | | [in 3] |
| **Hygromiidae** | | | | | | | | | |
| | **Hygromiinae** | **Hygromiini** | | | | | | | |
| | | | *Hygromia cinctella* (Draparnaud, 1801) | | Europe | 21 | 42 | | [in 3] |
| | | | *Hygromia limbata* (Draparnaud, 1805) | | Spain | 21 | 42 | | [in 4] |
| | | **Perforatellini** | | | | | | | |
| | | | *Monachoides incarnatus* (O. F. Müller, 1774) | *Perforatella incarnata* | Europe | 24 | 48 | | [in 3] |
| | **Leptaxinae** | **Leptaxini** | | | | | | | |
| | | | *Portugala inchoata* (Morelet, 1845) | | Portugal | 26 | 52 | | [in 4] |
| | | **Cryptosaccini** | | | | | | | |
| | | | *Mengoana jeschaui* (Kobelt, 1878) | | Spain | 26 | 52 | | [in 3] |
| | | | *Mengoana jeschaui* (Kobelt, 1878) | Eumphalia brigantina | Spain | 26 | 52 | | [11] |
| | | | *Pyrenaearia cantabrica poncebensis* Ortiz de Zárate | | Spain | 26 | 52 | | [in 4] |
| | | | *Pyrenaearia cantabrica poncebensis* Ortiz de Zárate | *Pyrenaearia poncebensis* | Spain | 26 | 52 | 13m, 8sm, 2st, 3t | [9] |
| | **Trochulininae** | **Caucasigenini** | | | | | | | |
| | | | *Caucasigena eichwaldi* (L. Pfeiffer, 1846) | | Georgia | 23 | 46 | | [12] |
| | | | *Circassina frutis (L. Pfeiffer, 1859)* | | Georgia | 23 | 46 | | [15] |
| | | | *Fruticocampylaea narzanensis* (Krynicki, 1836) | | Georgia | 23 | 46 | | [12] |
| | | **Monachaini** | | | | | | | |
| | | | *Diplobursa pisiformis arpatschaiana* (Mousson, 1873) | *Euomphalia arpatshuana* | Iran | 23 | 46 | | [in 3] |
| | | | *Euomphalia strigella* (Draparnaud, 1801) | | Spain | 26 | 48 | 19m,5sm | [9] |
| | | | *Monacha cantiana* (Montagu, 1803) | | Europe | 23 | 46 | | [in 3] |
| | | | *Monacha cartusiana* (O.F. Müller, 1774) | | Europe | 23 | 46 | | [in 3] |
| | | | *Monacha cartusiana* (O.F. Müller, 1774) | | Spain | 23 | 46 | 22m, 1sm | [9] |
| | | | *Monacha* sp. | | Italy | 23 | 46 | 20m, 3sm | this study |
| | | **Trochulini** | | | | | | | |
| | | | *Petasina unidentata* (Draparnaud, 1805) | *Trichia unidentata* | Europe | 23 | 46 | | [in 3] |
| | | | *Trochulus hispidus* (Linnæus, 1758) | *Trichia hispida* | Spain | 23 | 46 | | [11] |
| | | | *Trochulus hispidus* (Linnæus, 1758) | | Czech | 23 | 46 | | [19] |
| | | | *Trochulus plebeius* (Draparnaud, 1805) | | Czech | 23 | 46 | | [19] |
| | | | *Trochulus sericeus (Draparnaud, 1801)* | *Trichia sericea* | Europe | 23 | 46 | | [in 3] |
| | | | *Trochulus villosus* (Draparnaud, 1805) | *Trichia villosa* | Europe | 23 | 46 | | [in 3] |
| **Polygyridae** | | | | | | | | | |
| | **Triodopsinae** | **Ashmunellini** | | | | | | | |
| | | | *Ashmunella ashmuni ashmuni* (Dall, 1897) | | USA | 29 | 58 | | [20] |

| | | | | | |
|---|---|---|---|---|---|
| *Ashmunella levettei* (Bland, 1882) | | USA | 29 | 58 | [20] |
| *Ashmunella mendax* Pilsbry & Ferriss, 1917 | | USA | 29 | 58 | [20] |
| *Ashmunella mogollonensis* Pilsbry, 1905 | | USA | 29 | 58 | [20] |
| *Ashmunella carlsbadensis* Pilsbry, 1932 | | USA | 29 | 58 | [20] |
| *Ashmunella ferrissi* Pilsbry, 1905 | | USA | 29 | 58 | [20] |
| *Ashmunella kocki* Pilsbry, 1940 | | USA | 29 | 58 | [20] |
| *Ashmunella organensis* Pilsbry, 1936 | | USA | 29 | 58 | [20] |
| *Ashmunella pasonis* (Drake, 1951) | | USA | 29 | 58 | [20] |
| *Ashmunella rhissa altissima* (Cockerell, 1898) | | USA | 29 | 58 | [20] |
| *Ashmunella* sp *(ryssa complex)* | | USA | 29 | 58 | [20] |
| *Ashmunella tetrodon inermis* H. A. Pilsbry and J. H. | | USA | 29 | 58 | [20] |
| *Ashmunella thomsoniana* (Ancey, 1887) | | USA | 29 | 58 | [20] |
| *Ashmunella walkeri* Ferriss, 1904 | | USA | 29 | 58 | [20] |
| **Mesodontini** | | | | | |
| *Inflectarius inflectus* (Say, 1821) | | USA | 29 | 58 | [21] |
| *Inflectarius rugeli* (Shuttleworth, 1852) | *Mesodon rugeli* | USA | 29 | 58 | [21] |
| *Inflectarius sp.* Pilsbry, 1940 | | USA | 29 | 58 | [21] |
| *Mesodon andrewsae normalis* (W.G. Binney, 1879) | | USA | 29 | 58 | [21] |
| *Mesodon appressus* (Say, 1821) | | USA | 29 | 58 | [21] |
| *Mesodon mitchellianus* (I. Lea, 1838) | | USA | 29 | 58 | [21] |
| *Mesodon normalis* (Pilsbry, 1900) | | USA | 29 | 58 | [21] |
| *Mesodon thyroidus* (Say, 1817) | | USA | 29 | 58 | [in 3] |
| *Mesodon thyroidus* (Say, 1817) | | USA | 29 | 58 | [21] |
| *Mesodon zaletus* (A. Binney, 1837) | | USA | 29 | 58 | [21] |
| *Patera appressa* (Say, 1821) | *Polygyra appressa* | USA | 29 | 58 | [21] |
| *Patera appressa* (Say, 1821) | *Polygyra appressa* | USA | 31 | 62 | [in 21] |
| **Stenotrematini** | | | | | |
| *Stenotrema hirsutum* (Say, 1817) | | USA | 29 | 58 | [in 3] |
| *Stenotrema monodon aliciae* (Pilsbry, 1893) | | USA | 29 | 58 | [21] |
| *Stenotrema monodon aliciae* (Pilsbry, 1893) | | USA | 29 | 58 | [21] |
| *Stenotrema monodon aliciae* (Pilsbry, 1893) | *Stenotrema leai aliciae* | USA | 29 | 58 | [in 3] |
| *Stenotrema spinosum* (I. Lea, 1831) | | USA | 29 | 58 | [in 3] |
| *Stenotrema stenotrema* (L. Pfeiffer, 1842) | | USA | 29 | 58 | [in 3] |
| **Allogonini** | | | | | [in 3] |
| *Allogona profunda* (Say, 1821) | | USA | 26 | 52 | [in 3] |
| *Allogona townsendiana* (I. Lea, 1838) | | USA | 26 | 52 | [in 3] |
| *Cryptomastix germana* (Gould in W.G. Binney, 1851) | *Triodopsis germana* | USA | 31 | 62 | [in 3] |

| | | | | | | | | | |
|---|---|---|---|---|---|---|---|---|---|
| | | **Triodopisini** | | | | | | | |
| | | *Neohelix albolabris* (Say, 1817) | *Triodopsis albolabris* | USA | 29 | 58 | | | [21] |
| | | *Neohelix dentifera* (A. Binney, 1837) | *Triodopsis dentifera* | USA | 29 | 58 | | | [21] |
| | | *Triodopsis fraudolenta fraudolenta* (Pilsbry, 1894) | | USA | 29 | 58- | | | [21] |
| | | *Triodopsis fraudulent*a *vulgata* (Pilsbry, 1894) | | USA | 29 | 58- | | | [21] |
| | | *Triodopsis juxtidens juxtidens*(Pilsbry, 1894) | | USA | 29 | 58 | | | [21] |
| | | *Triodopsis tridentata* (Say, 1817) | | USA | 29 | 58 | | | [21] |
| | | *Triodopsis tridentata (Say, 1817)* | | USA | 29 | 58 | | | [21] |
| | | *Triodopsis tridentata. edentilabris* Pilsbry, 1940? | | USA | 29 | 58 | | | [21] |
| | | *Triodopsis vulgata* Pilsbry, 1940 | | USA | 29 | 58 | | | [21] |
| | | *Xolotrema fosteri* (F.C. Baker, 1932) | | USA | 31 | 62 | 21m, 7sm, 3st | NOR | [22] |
| | | **Vespericolini** | | | | | | | |
| | | *Vespericola columbiana* (I. Lea, 1839) | | USA | 30 | 60 | | | [in 3] |
| | **Polygyrinae** | | | | | | | | |
| | | *Polygira cereolus* (Megerle von Mühlfeldt, 1818) | | USA | 29 | 58 | | | [in 3] |
| **Trissexodontidae** | | | | | | | | | |
| | **Trissexodontinae** | **Oestophorini** | | | | | | | |
| | | *Oestophora barbula* (Rossmässler, 1838) | | Portugal | 30 | 60 | | | [in 4] |
| **Xanthonychidae** | | | | | | | | | |
| | **Helminthoglyptinae** | **Sonorellini** | | | | | | | |
| | | *Sonorella odorata* Pilsbry & Ferriss, 1919 | | USA | 29 | 58 | 15m, 13sm, 1st | | [23] |
| | | **Helminthoglyptini** | | | | | | | |
| | | *Eremarionta indioensis* (Yates, 1890) | | USA | 30 | 60 | 18m, 11sm, 1st | | [23] |
| | | *Greggelix indigena* (Mabille, 1895) | | USA | 29 | 58 | 16m, 10sm, 3st | | [23] |
| | | *Helminthoglypta cfr. lowei* (Bartsch, 1918) | | USA | 30 | 60 | 17m, 10sm, 3st | | [23] |
| | | *Helminthoglypta micrometalleoides* W.B. Miller, 1970 | | USA | 30 | 60 | 17m, 10sm, 3st | | [23] |
| | | *Micrarionta beatula* Cockerell, 1929 | *M. rufocincta beatula* | USA | 30 | 60 | 18m, 10sm, 2st | | [23] |
| | | *Sonorelix borregoensis* (S.S. Berry, 1929) | | USA | 29 | 58 | 13m, 15sm, 1st | | [23] |
| | | *Xerarionta areolata* (L. Pfeiffer, 1845) | | USA | 30 | 60 | 19m, 10sm, 3st | | [23] |
| | **Humboldtianinae** | | | | | | | | |
| | | **Humboldtianini** | | | | | | | |
| | | *Humboldtiana hogeana* (E. von Martens, 1892) | | USA | 30 | 60 | 16m, 11sm, 3st | | [23] |
| | **Monadeniinae** | | | | | | | | |
| | | *Monadenia fidelis* (J.E. Gray, 1834) | | USA | 29 | 58 | | | [in 23] |
| | | *Monadenia infumata* (Gould, 1855) | | USA | 29 | 58 | 16m, 12sm, 1st | | [23] |

## References


1. WoRMS Editorial Board. *World Register of Marine Species*. **2021**. http://www.marinespecies.org (last accessed June 15, 2021)
2. Molluscabase, **2021**. http,//www.molluscabase.org. (last accessed June 15, 2021)
3. Patterson, C.M. Chromosomes of molluscs. Proceedings of the 2nd Symposium of Mollusca, Ernakulam, Cochin, India. Marine Biological Association of India. Mandapam Camp. **1969**. Symposium series 3, part 2, 635-686.
4. Thiriot-Quiévreux, C. Advances in chromosomal studies of gastropod molluscs. *J. Molluscan Stud.* **2003**, 69, 187-201. DOI:10.1093/mollus/69.3.187.
5. Babrakzai, N.; Samsam, S.; Miller, W.B. Cytological Relationships of Helminthoglyptidae and Bradybaenidae (Mollusca, Pulmonata). *Proceedings of the Twenty-Fourth Annual Meeting of the Arizona-Nevada Academy of Science and The Fifty-Sixth Annual Meeting Southwestern and Rocky Mountain Division American Association for the Advancement of Science April 9 To April 12, 1980 MGM Hotel, Las Vegas, Nevada*, pp 31.
6. Park, G.M. Karyological analysis of Korean endemic land snail, *Koreanohadra kurodana* (Gastropoda: Bradybaenidae). *Caryologia* **2016**, *69*, 339-342. https://doi.org/10.1080/00087114.2016.1224403
7. Tatewaki, R.; Kitada, J. Karyological studies of five species of land snails (Helicoidea, Mollusca). *Genetica* **1987**, *74*, 73-80. https://doi.org/10.1007/BF00055097
8. Laws, H.M. The Chromosomes of Some Australian Camaenid Land Snails. *Cytologia* **1973**, *38*, 229-235. doi: 10.1508/cytologia.38.229.
9. Aparicio, M.T. Cytotaxonomic studies of the family Helicidae (Gastropoda, Eupulmonata). *Genét. Ibér*. **1981**, *33*, 211-224.
10. Vitturi, R.; Rasotto, M.B.; Farinella-Ferruzza, N. The chromosomes of 16 molluscan species. *Boll. Zool.* **1982**, *49*, 61-71. https://doi.org/10.1080/11250008209439373.
11. Ramos, M.A.; Aparicio, M.T. Cytotaxonomic Study of Some Spanish and Portuguese Helicidae (Pulmonata: Geophila). *Malocologícal Review* **1985**, *18*, 73 -82.
12. Chakvetadze, N.; Bakhtadze N.; Mumladze, L.; Tskhadaia, E. Chromosome numbers of terrestrial mollusks (Mollusca: Gastropoda) of Georgia. 1st Meeting of the Mollusc Research Society Austria (MoFA) in Salzburg. *Arianta* **2019**, *7*, 10.
13. Bantock, C.R. Localisation of chiasmata in *Cepaea nemoralis* L. *Heredity* **1972**, *29*, 213-221. https://doi.org/10.1038/hdy.1972.85.
14. Page, C. The Karyotype of the Land Snail *Cepaea Nemoralis* (L.). *Heredity* **1978**, *41*, 321-325. https://doi.org/10.1038/hdy.1978.102.
15. Bakhtadze, N.G.; Chakvetadze, N.L.; Mumladze, L.J.; Bakhtadze, G.I.; Tskhadaia, E.A. Karyological data of terrestrial mollusks (Mollusca: Gastropoda: Pulmonata) of Georgia. *Proc. Inst. Zool.* **2014**, 25, 23-27. DOI:10.13140/2.1.4677.1525
16. Petraccioli, A.; Crovato, P.; De Vico, G.; Odierna, G.; Picariello, O.L.A.; Tardy, E.; Viglietti, S.; Guarino, F.M.; Maio, N. *Helix straminea* Briganti, 1825 in Italy (Gastropoda, Pulmonata), taxonomic history, morphology, biology, distribution and phylogeny. *Eur. Zool. J.* **2021**, *88*, 390–416, doi:10.1080/24750263.2021.1892217.

17. Vitturi, R.; Libertini, A.; Sineo, L.; Sparacio, I.; Lannino, A.; Gregorini, A.; Colomba, M. Cytogenetics of the land snails *Cantareus aspersus* and *C. mazzullii* (Mollusca, Gastropoda, Pulmonata). *Micron* **2005**, 36, 351-357. DOI: 10.1016/j.micron.2004.12.010

18. Aparicio, M.T. The chromosomes of eight species of the subfamilv Helicinae (Gastropoda, Pulmonata, Helicidae) from Spain. *Malacological Review* **1983**, *16*, 71-78.

19. Hrabakova, M.; Juricková, L.; Petrusek, A. Taxonomy of the genus *Trochulus* (Gastropoda: Hygromiidae) in the Czech Republic. *Abstracts of Molluscan Forum November 2nd 2006 held in the Palaeontology Demonstration Room of the Natural History Museum, London*. *The Malacologist* **2006**.

20 Stern, E.M.; Metcalf, A.L. Chromosome Numbers in *Ashmunella* (Gastropoda Eupulmonata Polygyridae). *Veliger* **1974**, *17*, 19- 22.

21. Husted, L.; Burch, P.R. The Chromosomes of Polygyrid Snails. *Am. Nat.* **1946**, *80*, 410-429.

22.Diopotex-Chong, M.E.; Babrakzai, N. Preliminary cytological studies in *Triodopsis fosteri* (Baker, 1920) Mollusca-Eupulmonata Polygiridae. *An. Inst. Cienc. del Mar y Limnol*. **1993**, *19*, 13-18.

23. Babrakzai, N. Chromosomes of Helminthoglyptidae (Pulmonata: Gastropoda). PhD dissertation **1975**, Department of Biological Sciences, University of Arizona.

**Table S2**. Relative length (RL) and Centromeric Index (CI) of the studied taxa. Mo. Sp. = *Monacha* sp.; Tr.e. = *Trochoidea elegans*; Tr.p. = *Trochoidea pyramidata*; Tr.t. = *Trochoidea trochoides*; Ce.v. = *Cernuella virgata*; Co.a. = *Cochlicella acuta*; Eo.v. = *Eobania vermiculata*; Ot.l. = *Otala lactea*; Co.ap. = *Cornu apertus*; Er.m. = *Erctella mazzullii*; He.g. = *Helix gussoneana*; He.l. = *Helix lucorum*; Th.p. A = *Theba pisana* (Fusaro, Naples); Th.p. B = *Theba pisana* (Messina); Ca.p. = *Campylaea planospira*; Ma.. = *Marmorana platychela*.

| | **Mo. sp.** | | **Tr.e.** | | **Tr.p-** | | **Tr.t.** | | **Ce.v.** | | **Co.a.** | | **Eo.v.** | | **Ot.l** | | **Co.ap.** | | **Er.m.** | | **He.g.** | | **He.l.** | | **Th.p. A** | | **Th. p. B** | | **Ca.p.** | | **Ma.p.** | |
|---|---|---|---|---|---|---|---|---|---|---|---|---|---|---|---|---|---|---|---|---|---|---|---|---|---|---|---|---|---|---|---|---|
| **Chr.** | **RL** | **CI** | **RL** | **CI** | **RL** | **CI** | **RL** | **CI** | **RL** | **CI** | **RL** | **CI** | **RL** | **CI** | **RL** | **CI** | **RL** | **CI** | **RL** | **CI** | **RL** | **CI** | **RL** | **CI** | **RL** | **CI** | **RL** | **CI** | **RL** | **CI** | **RL** | **CI** |
| **1** | 9.3 | 42.7 | 7.5 | 37.1 | 8.3 | 30.5 | 7.9 | 35.4 | 9.5 | 45.2 | 10.9 | 48.8 | 12.3 | 44.4 | 11.7 | 47.9 | 7.5 | 47.8 | 7.2 | 44.6 | 9.5 | 47.5 | 7.9 | 38.4 | 7.2 | 46.4 | 7.0 | 44.5 | 6.2 | 46.5 | 5.9 | 46.7 |
| **2** | 8.0 | 34.9 | 6.9 | 47.2 | 8.0 | 45.1 | 7.4 | 42.8 | 6.1 | 39.8 | 9.9 | 43.5 | 6.9 | 41.0 | 6.6 | 47.5 | 5.2 | 41.8 | 5.0 | 41.6 | 7.4 | 40.1 | 6.5 | 31.3 | 5.2 | 39.6 | 5.0 | 41.8 | 5.1 | 47.4 | 5.6 | 48.1 |
| **3** | 6.9 | 44.4 | 6.7 | 39.8 | 7.3 | 40.2 | 7.0 | 36.5 | 4.7 | 41.9 | 6.1 | 48.1 | 6.3 | 40.9 | 6.3 | 45.2 | 5.0 | 44.4 | 4.9 | 42.7 | 5.8 | 43.2 | 6.5 | 10.9 | 4.5 | 48.0 | 4.6 | 45.8 | 4.6 | 41.9 | 5.2 | 45.5 |
| **4** | 5.7 | 40.1 | 4.5 | 39.7 | 5.2 | 43.1 | 5.1 | 31.7 | 4.5 | 44.0 | 5.5 | 46.1 | 5.1 | 41.1 | 4.9 | 43.5 | 4.7 | 41.7 | 4.8 | 42.8 | 5.2 | 48.2 | 5.2 | 47.8 | 4.4 | 46.1 | 4.5 | 43.5 | 4.4 | 44.3 | 5.1 | 45.9 |
| **5** | 4.8 | 40.6 | 4.5 | 39.6 | 5.0 | 34.7 | 5.1 | 47.3 | 4.1 | 40.6 | 5.0 | 39.4 | 4.7 | 43.5 | 4.6 | 45.3 | 4.6 | 24.7 | 4.5 | 45.9 | 4.8 | 42.9 | 5.1 | 0.0 | 4.4 | 43.8 | 4.3 | 41.1 | 4.2 | 43.6 | 4.7 | 47.4 |
| **6** | 4.8 | 39.5 | 4.4 | 44.9 | 4.5 | 36.6 | 4.6 | 34.1 | 4.1 | 41.9 | 4.9 | 44.7 | 4.7 | 46.1 | 4.3 | 49.0 | 4.4 | 43.6 | 4.3 | 43.8 | 4.3 | 42.1 | 5.1 | 10.0 | 4.1 | 43.1 | 4.0 | 42.5 | 4.0 | 48.6 | 4.2 | 38.8 |
| **7** | 4.4 | 41.8 | 4.1 | 43.4 | 4.4 | 47.8 | 4.6 | 47.0 | 4.0 | 41.9 | 3.9 | 35.8 | 4.4 | 42.3 | 3.8 | 43.7 | 4.4 | 44.8 | 4.2 | 39.2 | 4.3 | 29.1 | 5.0 | 0.0 | 3.9 | 45.6 | 3.9 | 46.9 | 4.0 | 44.9 | 4.1 | 40.8 |
| **8** | 4.4 | 42.2 | 4.1 | 34.7 | 4.3 | 41.2 | 4.1 | 29.5 | 4.0 | 45.6 | 3.9 | 35.6 | 4.0 | 41.8 | 3.8 | 31.3 | 4.1 | 41.8 | 4.0 | 40.3 | 4.2 | 48.3 | 4.4 | 8.8 | 3.6 | 44.0 | 3.7 | 43.0 | 4.0 | 38.9 | 4.0 | 40.6 |
| **9** | 4.4 | 40.3 | 4.1 | 31.9 | 4.3 | 39.4 | 4.0 | 43.8 | 3.9 | 45.1 | 3.9 | 41.5 | 3.9 | 45.5 | 3.8 | 37.1 | 4.0 | 36.2 | 3.9 | 44.2 | 4.1 | 42.1 | 4.1 | 9.9 | 3.4 | 39.7 | 3.6 | 40.6 | 4.0 | 43.5 | 3.9 | 46.2 |
| **10** | 4.2 | 44.8 | 4.0 | 45.4 | 3.9 | 40.6 | 3.9 | 40.3 | 3.9 | 43.2 | 3.7 | 8.8 | 3.8 | 46.3 | 3.8 | 40.0 | 4.0 | 48.3 | 3.9 | 44.4 | 4.4 | 47.2 | 4.0 | 41.2 | 3.4 | 40.8 | 3.5 | 44.2 | 3.9 | 44.7 | 3.9 | 38.2 |
| **11** | 4.1 | 48.4 | 4.0 | 7.8 | 3.8 | 32.5 | 3.8 | 31.5 | 3.9 | 40.4 | 3.6 | 47.5 | 3.6 | 40.7 | 3.7 | 42.9 | 4.0 | 47.2 | 3.9 | 42.9 | 4.0 | 46.6 | 3.8 | 47.0 | 3.3 | 47.8 | 3.5 | 46.1 | 3.6 | 48.6 | 3.6 | 39.9 |
| **12** | 4.0 | 45.9 | 4.0 | 36.9 | 3.7 | 38.5 | 3.8 | 39.7 | 3.9 | 41.3 | 3.5 | 33.2 | 3.4 | 34.2 | 3.4 | 34.8 | 3.9 | 41,3 | 3.8 | 43.0 | 3.3 | 33.7 | 3.6 | 0.0 | 3.3 | 46.8 | 3.4 | 44.4 | 3.4 | 44.2 | 3.5 | 46.5 |
| **13** | 4.2 | 34.0 | 4.0 | 41.0 | 3.7 | 40.2 | 3.8 | 40.4 | 3.6 | 32.3 | 3.4 | 41.1 | 3.3 | 38.4 | 3.4 | 44.3 | 3.6 | 43.4 | 3.8 | 42.2 | 3.2 | 24.3 | 3.3 | 0.0 | 3.3 | 38.6 | 3.2 | 40.6 | 3.4 | 46.8 | 3.4 | 43.1 |
| **14** | 4.0 | 43.2 | 4.0 | 42,6 | 3.4 | 47.6 | 3.7 | 44.9 | 3.6 | 42.3 | 3.3 | 39.1 | 3.3 | 47.4 | 3.3 | 40.6 | 3.4 | 42.3 | 3.7 | 39.5 | 3.1 | 34.3 | 3.2 | 38.6 | 3.2 | 43.2 | 3.2 | 40.3 | 3.3 | 47.6 | 3.2 | 48.8 |
| **15** | 3.9 | 43.1 | 3.8 | 40.8 | 3.3 | 42.4 | 3.7 | 38.6 | 3.6 | 32.9 | 3.2 | 39.5 | 2.9 | 45.5 | 3.3 | 38.1 | 3.4 | 46.0 | 3.4 | 44.1 | 3.1 | 39.9 | 3.0 | 0.0 | 3.2 | 44.5 | 3.2 | 43.2 | 3.3 | 44.6 | 3.1 | 39.8 |
| **16** | 3.7 | 41.6 | 3.7 | 27.2 | 3.3 | 46.0 | 3.7 | 43.8 | 3.3 | 40.0 | 3.2 | 27.9 | 2.9 | 43.6 | 3.2 | 44.1 | 3.3 | 46.5 | 3.4 | 31.0 | 2.9 | 47.1 | 2.7 | 40.9 | 3.1 | 44.2 | 3.1 | 44.9 | 3.2 | 39.5 | 2.9 | 42.8 |
| **17** | 3.3 | 40.8 | 3.6 | 36.4 | 3.3 | 44.8 | 3.5 | 40.0 | 3.2 | 38.6 | 2.9 | 23.6 | 2.9 | 40.4 | 3.2 | 30.1 | 3.2 | 40.5 | 3.3 | 48.4 | 2.9 | 26.8 | 2.7 | 7.9 | 3.1 | 41.1 | 3.1 | 39.1 | 3.2 | 43.5 | 2.9 | 39.5 |
| **18** | 3.0 | 41.9 | 3.6 | 42,3 | 3.3 | 44.8 | 3.3 | 29.1 | 3.2 | 39.0 | 2.6 | 37.6 | 2.7 | 43.9 | 3.1 | 45.5 | 3.2 | 29.8 | 3.1 | 45.4 | 2.9 | 46.9 | 2.7 | 0.0 | 3.0 | 47.2 | 3.0 | 48.0 | 2.8 | 41.9 | 2.8 | 43.8 |
| **19** | 2.6 | 43,8 | 3.5 | 44.7 | 3.1 | 45.4 | 3.3 | 40.2 | 3.1 | 39.1 | 2.6 | 43.2 | 2.6 | 42.9 | 3.0 | 44.3 | 3.1 | 40.7 | 3.0 | 39.8 | 2.8 | 42.2 | 2.6 | 0.0 | 2.9 | 45.6 | 2.9 | 46.1 | 2.8 | 44.9 | 2.8 | 44.2 |
| **20** | 3.7 | 39.9 | 3.4 | 41.9 | 2.9 | 40.0 | 3.2 | 42.9 | 3.1 | 40.1 | 2.3 | 47.3 | 2.6 | 41.1 | 2.9 | 35.8 | 3.1 | 42.3 | 3.0 | 32.8 | 2.7 | 48.8 | 2.6 | 0.0 | 2.8 | 41.8 | 2.9 | 40.0 | 2.7 | 44.1 | 2.7 | 40.2 |
| **21** | 2.6 | 40.9 | 3.2 | 29.7 | 2.9 | 47.9 | 3.1 | 39,4 | 3.0 | 39.3 | 2.1 | 44.6 | 2.6 | 45.7 | 2.9 | 48.7 | 2.9 | 39,6 | 3.0 | 47.8 | 2.5 | 45.9 | 2.5 | 45.8 | 2.8 | 42.4 | 2.8 | 40.4 | 2.7 | 43.5 | 2.7 | 42.5 |
| **22** | 2.3 | 32.2 | 3.2 | 46.6 | 2.9 | 43.9 | 2.6 | 40.3 | 2.8 | 45.5 | 2.1 | 18.1 | 2.6 | 29.8 | 2.8 | 39.5 | 2.6 | 41.9 | 3.0 | 41.6 | 2.4 | 41.2 | 2.5 | 45.8 | 2.7 | 39.8 | 2.7 | 41.9 | 2.7 | 40.4 | 2.6 | 29.4 |
| **23** | 1.7 | 42.2 | 2.8 | 0.0 | 2.8 | 43.8 | 2.5 | 38.1 | 2.8 | 38.2 | 2.0 | 32.7 | 2.5 | 45.5 | 2.8 | 38.9 | 2.6 | 36.0 | 2.9 | 42.3 | 2.3 | 48.8 | 2.5 | 0.0 | 2.6 | 46.7 | 2.6 | 45.8 | 2.7 | 42.8 | 2.5 | 42.0 |
| **24** | | | 2.6 | 38.6 | 2.3 | 46.3 | 2.2 | 0.0 | 2.8 | 43.7 | 1.9 | 42.2 | 2.3 | 44.3 | 2.4 | 40.8 | 2.5 | 40.6 | 2.6 | 45.0 | 2.1 | 40.1 | 2.4 | 0.0 | 2.5 | 44.6 | 2.5 | 46.0 | 2.7 | 45.6 | 2.4 | 46.2 |
| **25** | | | | | | | | | 2.6 | 46.1 | 1.9 | 40.5 | 2.0 | 45.9 | 2.0 | 42.3 | 2.5 | 31.7 | 2.6 | 42.7 | 2.1 | 43.4 | 2.2 | 0.0 | 2.5 | 40.1 | 2.5 | 42.6 | 2.6 | 46.0 | 2.3 | 42.8 |
| **26** | | | | | | | | | 2.6 | 46.4 | 1.8 | 41.3 | 2.0 | 46.8 | 1.1 | 0.0 | 2.4 | 43.0 | 2.6 | 40.4 | 2.1 | 41.8 | 2.2 | 46.5 | 2.3 | 40.0 | 2.4 | 39.9 | 2.4 | 41.4 | 2.2 | 40.5 |
| **27** | | | | | | | | | | | | | | | | | 2.3 | 42.6 | 2.4 | 45.8 | 1.9 | 20.0 | 1.6 | 48.3 | 2.3 | 45.1 | 2.3 | 430 | 2.3 | 46.2 | 2.2 | 41.0 |
| **28** | | | | | | | | | | | | | | | | | | | | | | | | | 2.3 | 42.2 | 2.3 | 41.0 | 2.3 | 31.9 | 2.1 | 38.1 |
| **29** | | | | | | | | | | | | | | | | | | | | | | | | | 2.3 | 48.6 | 2.2 | 44.2 | 1.8 | 45.4 | 1.8 | 29.8 |
| **30** | | | | | | | | | | | | | | | | | | | | | | | | | 2.3 | 48.5 | 2.2 | 45.9 | 1.7 | 43.7 | 1.6 | 49.0 |

**Table S3.** Distance matrix of the homologous traits of 16S rDNA of the *Monacha* species considered in the paper. M. cart = *Monacha cartusiana*; M. cisc = *Monacha ciscaucasica*; IT = Italy; Bosn Herz = Bosnia Herzegovina.

| | | | | | | | | | | | |
|---|---|---|---|---|---|---|---|---|---|---|---|
| GAST 32 Portici (IT) | 0.0000 | 0.1737 | 0.1737 | 0.1737 | 0.1689 | 0.1737 | 0.1689 | 0.1580 | 0.1624 | 0.1691 | 0.1898 |
| M. cart KM247397 Brescia (IT) | 0.1737 | 0.0000 | 0.0000 | 0.0000 | 0.0037 | 0.0000 | 0.0075 | 0.1894 | 0.1940 | 0.1413 | 0.1704 |
| M. cart KX495378 Lombardia (IT) | 0.1737 | 0.0000 | 0.0000 | 0.0000 | 0.0037 | 0.0000 | 0.0075 | 0.1894 | 0.1940 | 0.1413 | 0.1704 |
| M. cart AY741416 Siena (IT) | 0.1737 | 0.0000 | 0.0000 | 0.0000 | 0.0037 | 0.0000 | 0.0075 | 0.1894 | 0.1940 | 0.1413 | 0.1704 |
| M. cart M247391 Hungary | 0.1689 | 0.0037 | 0.0037 | 0.0037 | 0.0000 | 0.0037 | 0.0037 | 0.1847 | 0.1893 | 0.1368 | 0.1657 |
| M. cart MG585433 Spain | 0.1737 | 0.0000 | 0.0000 | 0.0000 | 0.0037 | 0.0000 | 0.0075 | 0.1894 | 0.1940 | 0.1413 | 0.1704 |
| M. cart MH204083 Bosn. Herz. | 0.1689 | 0.0075 | 0.0075 | 0.0075 | 0.0037 | 0.0075 | 0.0000 | 0.1847 | 0.1893 | 0.1368 | 0.1704 |
| M. cisc KX495397 Russia | 0.1580 | 0.1894 | 0.1894 | 0.1894 | 0.1847 | 0.1894 | 0.1847 | 0.0000 | 0.0181 | 0.1608 | 0.2030 |
| M. roseni KX495386 Russia | 0.1624 | 0.1940 | 0.1940 | 0.1940 | 0.1893 | 0.1940 | 0.1893 | 0.0181 | 0.0000 | 0.1608 | 0.2076 |
| M. nummus KX495427 Lebanon | 0.1691 | 0.1413 | 0.1413 | 0.1413 | 0.1368 | 0.1413 | 0.1368 | 0.1608 | 0.1608 | 0.0000 | 0.1864 |
| M. sp1 KX495425 Sicily (IT) | 0.1898 | 0.1704 | 0.1704 | 0.1704 | 0.1657 | 0.1704 | 0.1704 | 0.2030 | 0.2076 | 0.1864 | 0.0000 |
